# Extracellular vehicles-mediated Twsit1 transferred from tumor cells to brain induces depressive-like behaviors via neuronal morphogenesis

**DOI:** 10.7150/thno.112238

**Published:** 2025-08-16

**Authors:** Ruo-Si Zou, Jin-Gang He, Yang Zhao, Bing Zhou, Si-Long Deng, Jian-Guo Chen, Fang Wang

**Affiliations:** 1 Department of Pharmacology, School of Basic Medicine and Department of Pharmacy, Tongji Hospital, Tongji Medical College, and State Key Laboratory for Diagnosis and Treatment of Severe Zoonotic Infectious Diseases, Huazhong University of Science and Technology, Wuhan, Hubei, 430030, China.; 2 The Key Laboratory for Drug Target Researches and Pharmacodynamic Evaluation of Hubei Province, Wuhan, Hubei, 430030, China.; 3 The Research Center for Depression, Tongji Medical College, Huazhong University of Science and Technology, Wuhan, Hubei, 430030, China.; 4 Hubei Shizhen Laboratory, Wuhan, Hubei, 430030, China.

**Keywords:** depression, tumor cell, extracellular vehicles (EVs), Twist1, dendritogenesis.

## Abstract

**Rationale:** Depression is commonly comorbid with cancer and affects therapeutic efficacy and outcome-of-disease. However, the molecular mechanism underlying cancer-induced depression (CID) remains poorly understood. Twist1 is a proto-oncogene driving tumor progression and metastasis, and chronic stress induces Twist1 expression in the medial prefrontal cortex (mPFC). This study aims to investigate the role and mechanisms of tumor-derived Twist1 in CID.

**Methods:** shTwist1 stably expressing 4T1 cells were obtained through lentivirus transduction and puromycin selection. Tumor cells were subcutaneously inoculated into mice to establish a tumor-bearing mice model. Behavioral assays were used to assess depressive-like behaviors in mice. Ultra-high-speed centrifugation was employed to extract extracellular vehicles (EVs) in 4T1 cell medium or serum from tumor-bearing mice. Quantitative polymerase chain reaction and western blot were used to detect the levels of Twist1 mRNA and protein from tumor-derived EVs or mPFC tissue. Lentivirus was injected into the mPFC to knock down Twist1. Intravenous or intranasal administration of tumor or serum-derived EVs were used to investigate the role of EVs-packaged Twist1 in depressive-like behaviors in mice.

**Results:** The present study demonstrated that tumor-derived EVs mediated the inter-organ communication between tumor cells and brain. Pharmacological inhibition of EVs secretion mitigated depressive-like behaviors in tumor-bearing mice. Intravenous or intranasal injection of EVs from tumor cells or serum from tumor-bearing mice into naïve mice induced a depressive-like phenotype. Further investigation identified tumor-derived EVs Twsit1 as a crucial mediator of cancer-induced dendritic atrophy and depressive-like behaviors in tumor-bearing mice. Knockdown of Twist1 in tumor cells significantly alleviated the detrimental effects of tumor-derived EVs on neuronal morphogenesis and prevented their pro-depressant effects.

**Conclusions:** This study demonstrates that tumor-derived EVs containing Twist1 constitute a key pathological driver of cancer-induced depression, revealing a potential therapeutic target for clinical intervention.

## Introduction

Major depressive disorder ranks among the leading causes of global disability, affecting over 260 million individuals worldwide 1. Notably, the prevalence of depressive disorders in cancer patients is three to five times higher than that in the general population 2. Persistent psychological distress, particularly comorbid depression or anxiety in cancer patients, exacerbates treatment burden and impedes disease management efficacy 3. Depression significantly elevates mortality risks in chronic medical conditions such as cancer. Evidence-based interventions targeting depression demonstrate improved survival outcomes, particularly among women with metastatic malignancies 4-6. Although chronic stress has been linked to carcinogenesis via hypothalamus-pituitary-adrenal axis dysfunction and sympathetic nervous system activation 7-9, the mechanistic link between cancer pathology and depression occurrence remains poorly understood. Conventional antidepressants (e.g., tricyclic anti-depressants and selective serotonin reuptake inhibitors) exhibit limited efficacy in treating cancer-induced depression (CID) 10, 11, underscoring the need for mechanistic investigations into CID.

Twist, a basic helix-loop-helix transcription factor, regulates cell migration and invasion during embryonic development and has been implicated in both embryogenesis and carcinogenesis 12. It is also overexpressed in diverse invasive carcinomas, including breast, prostate, esophageal, gastric, hepatic, pancreatic, and bladder cancers. While the underlying mechanisms differ, Twist1 involved in multiple oncogenic processes, including cancer cell senescence, apoptosis evasion, chemoresistance, differentiation blockade, invasiveness, and metastasis, with its pro-metastatic effects primarily mediated through epithelial-mesenchymal transition induction 13, 14. Despite extensive characterization of its pro-oncogenic roles, Twist1's functions within neurological contexts remain poorly elucidated. While normally expressed at low levels in mature neurons, Twist1 is upregulated in mutant huntingtin-expressing neurons and correlates with disease progression in the striatum and cortex of R6/2 mice, a widely used model for studying the pathogenesis of Huntington's disease 15, 16. Our recent study demonstrates that chronic stress triggers significant upregulation of Twist1 in the medial prefrontal cortex (mPFC), which subsequently disrupts dendritic arborization and drives depressive-like phenotypes in mice, suggesting a potential role for Twist1 in CID pathogenesis 17.

Extracellular vehicles (EVs) are membrane-bound vesicles secreted by mammalian cells, carrying microRNAs, nucleic acids, lipids, and proteins 18. The primary function of EVs lies in intercellular information transfer, influencing the cell physiology of recipient. Tumor-derived EVs released from the primary tumor cells enter the circulation, travel to distant organs, and reshape the local microenvironment in premetastatic organs to facilitate future metastasis 19, 20. Tumor-derived EVs have been shown to disrupt and cross the blood-brain barrier via transcytosis, promoting intracranial metastasis-particularly in breast cancer 21, 22. Correspondingly, EVs act as a novel mode of inter-neuronal communication, regulating physio-logical processes like synapse growth and plasticity 23, 24. Notably, blood-derived exosomes from depressed patients promote the occurrence of depression via miRNA delivery 25, while some non-brain-derived EVs (e.g., from bone marrow mesenchymal stem cells or natural killer cells) exhibit antidepressant effects 26, 27. These findings highlight context-dependent roles of EVs in depression. However, the potential role of tumor-derived EVs in depression pathogenesis remains unexplored.

In this study, we demonstrated that tumor-derived EVs contributed to CID via delivery of twist1. Exogenous administration of tumor-derived EVs or serum-derived EVs (SDEVs) from tumor-bearing mice induced depressive-like behaviors in naïve mice. Mechanistically, tumor-derived EVs-packaged Twist1 induced defective morphogenesis of dendrites of pyramidal neurons in the mPFC by inhibiting peroxisome proliferator-activated receptor δ (PPAR-δ). Our study elucidates the molecular mechanisms underlying CID, demonstrating how EVs deliver Twist1 to disrupt dendritic remodeling and thereby promote CID progression.

## Methods

### Cell culture

4T1, Lewis, MC38 cells used in this study were originally from American Type Culture Collection and cultured in the Dulbecco's Modified Eagle Medium (Gibco Laboratories, Grand Island, NY) supplemented with 10% fetal bovine serum, 100 U/mL of penicillin and 100 μg/mL of streptomycin. For exosome isolation from culture supernatants, cells were cultured in exosome-depleted fetal bovine serum 20. Cells were maintained in a humidified 37 °C incubator with 5% CO_2_. For the stable knockdown Twist1 in 4T1 cell lines, lentiviral vector expressing shRNA targeting Twist1 sequence GCTGAGCAAGATTCAGACC and puromycin resistance gene was designed by GeneChem company (Shanghai, China). After lentiviral infection and primary puromycin selection at 8 μg/mL, cell pools of 4T1/shTwist1 were obtained for 4-5 culture passages under puromycin concentration (5 μg/mL). For primary cultures of cortical neurons, cerebral cortices were prepared from C57BL/6 mice on postnatal days 0-3. The cerebral cortices were dissected, digested, and plated on coverslips. Neurons were cultured in neurobasal medium containing B27 supplement, 10% fetal bovine serum, penicillin/streptomycin mixture of antibiotics (100 U/mL) and glutamax (0.5 mM). 50 μL exosome (300 μg/mL) were added into the neurons at day 7 *in vitro* and the further analysis was carried out 7 days after administration.

### Animals

Adult female C57BL/6J mice (5 weeks old) were obtained from Hunan SJA Laboratory Animal Co., Ltd (Changsha, Hunan, China). All animals were habituated in our facility about 1 week before experiment and housed at consistent ambient temperature (21 ± 1 °C) and humidity (50% ± 5%) on a 12 h light/dark cycle with access to food and water *ad libitum*. All animal procedures were conducted in accordance with relevant guidelines and the institutional Animal Care and Use Committee of Huazhong University of Science and Technology.

### Inoculation of tumor cells

The inoculations of tumor cell were performed as previously described with minor modifications 28. Briefly, mice were anaesthetized with isoflurane, and then inoculated with 20,000 tumor cells (4T1, Lewis, MC38) in sterile phosphate-buffered saline (PBS) subcutaneously above their right back. Control mice were sham-inoculated with equal PBS. Tumor size was monitored using a digital caliper every 3 days once tumors became palpable at day 9. All behavioral tests were performed 21 days after cancer cell inoculation.

### EVs purification and characterization

EVs from cell culture medium or serum samples were purified by ultracentrifugation 20. Briefly, the culture medium was centrifuged at 500 g for 10 min and then at 12,000 g for 20 min. EVs were collected by ultracentrifugation of this supernatant at 100,000 g for 70 min, and the pellet was washed by resuspending in PBS and re-ultra centrifuging at 100,000 g for 70 min. EVs were resuspended in PBS and used in various experiments. Serum-derived EVs were separated using similar experimental approaches. Collected mouse blood samples were allowed to clot naturally at room temperature for 1 h, followed by centri-fugation at 2000 g for 10 min to obtain the supernatant serum. 500 μL serum was mixed with 3 mL of PBS, followed by centrifugation at 10000 g for 30 min. The supernatant was then used for subsequent ultracentrifugation. The EVs protein concentration was determined by bicinchoninic acid assay (Beyotime Biotechnology, Haimen, China).

### Transmission electron microscopy

Transmission electron microscopy was utilized to analyze morphological features of EVs. First, EV pellets were resuspended in PBS and applied as a drop onto Parafilm. A 200-mesh copper grid was placed over the exosome suspension for 2 min. Subsequently, the grid was negatively stained with 2% phosphotungstic acid for 5 min. Samples were imaged using electron microscopy (FEI Company, USA) operated at an accelerating voltage of 80 kV.

### Nanoparticle tracking analysis (NTA)

The NanoFCM U30E instrument (Xiamen, China) was used to determine the size distribution and concentration of the EVs. Prior to analysis, isolated EV samples were serially diluted with PBS buffer to achieve an optimal concentration for detection (e.g., targeting approximately 1×10^7^-1×10^9^ particles/mL, or within the instrument's linear range). Then, 100 μL of the diluted EVs suspension was loaded into the sample chamber. Data acquisition and subsequent analysis to determine size mode (nm) and particle concentration (particles/mL) were performed using NF Profession 2.0 software.

### EVs labelling and administration

EVs isolated from 4T1 cells were labeled with PKH67 (Sigma-Aldrich, St. Louis, MO, USA) according to the manufacturer's instructions. Briefly, 4T1 cells-derived EVs were first resuspended in diluent (500 µL), followed by addition of 2 μL PKH67 dye for 1 h of incubation at 37 °C in the dark. The labelled exosomes were subsequently washed with PBS, followed by centrifugation at 110,000 g and 4 °C for 70 min to remove the residual PKH67 dye. After washing with PBS and additional centrifugation, EVs were resuspended in PBS (300 μg/mL). Mice were injected intravenously with 50 μL PKH67-labelled exosomes and then intracardially perfused with paraformaldehyde 4 h later. Serial brain slices containing (30 μm) was obtained using a freezing microtome (CM1900, Leica Microsystems, Wetzlar, Germany). 4',6-diamidino-2-phenylindole was used for nuclear staining. The incorporation of PKH67-labelled exosomes into the brain was analyzed by laser scanning microscope (FV1000, Olympus, Tokyo, Japan). For EVs administration, intravenous and intranasal administration were used 29. EVs were isolated from serums or cell culture medium and then resuspended in PBS a concentration of 300 μg/mL for experiments. Recipient mice were subjected to tail vein injection of 50 μL EVs or intranasal injection of 25 μL EVs every 7 days. There were 3 injections in total. The number of EVs for intravenous injection and intranasal injection were approximately 2×10^10^ particles and 1×10^10^ particles, respectively. Intranasal administration was carried out with no more than 5 μL administered per nostril and 10 min of recovery between intranasal injections before moving to the opposite nostril.

### GW4869 and RNase treatment

GW4869 (#HY-19363, MCE, USA) was dissolved in dimethyl sulfoxide at 8 mg/mL. The solution was freshly prepared before use in PBS or 0.9% normal saline. *In vitro*, GW4869 was added to the exosome-depleted culture medium at a concentration of 10 μM. After culturing 4T1 cells for 24 h in medium containing GW4869, proteins were extracted from three sources: the cells themselves, exosomes in the culture medium, and the remaining supernatant after exosome isolation, for subsequent protein quantification and western blot analysis. *In vivo*, mice were injected with GW4869 (2.5 mg/kg, i.p) every 3 days from 10 to 21 days after subcutaneous inoculations of 4T1 cells. For RNase A treatment, purified SDEVs were incubated (37 °C, 30 min) with 2 mg/mL protease-free RNase A (TaKaRa, Dalian, China). SDEVs were also subjected to treatment with 0.1% Triton X-100 for 30 min at 37 °C after RNase A was added. After resuspension, EVs were used for subsequent quantitative reverse transcription-PCR experiments.

### Behavioral assessments

**Sucrose preference test (SPT).** Mice were acclimatized with two identical 50 mL bottles containing 1% sucrose or water alone (drinking water). The position of the bottles was interchanged every 24 h to eliminate position preference. After habituated for 48 h, mice were deprived of water for 20 h. For preference test, mice were exposed to bottles with 1% sucrose and water for ensuing 2 h (20:00-22:00 pm). The sucrose preference was calculated as the fraction of the sucrose solution compared to the total amount of consumed liquid.

**Open field test (OFT).** Spontaneous activity of mice was assessed with OFT. Animals were habituated in the test room ~1 h before the test. Mice were exposed to an 50 × 50 × 40 cm open field Plexiglas box. The total moving distance was recorded using ANY-maze software (Stoelting Co. USA) for 6 min.

**Tail suspension test (TST).** The tail suspension test was based on the described procedure as previously 30. Mice were suspended by adhering tail securely to the suspension bar with tape. Mice were suspended 20 cm above the floor with tape placed 15 cm from the tip of the tail. The immobility was defined when they exhibited no body movement and hung passively. The immobile time was quantified over the 6-min observation period.

**Forced swim test (FST).** The forced swim test was based on a previously described procedure 31. Mice were individually placed in the Plexiglas cylinders (25 cm height) containing 20 cm water (25 ± 1 ºC). Every session was videotaped and the water was changed after each session to avoid any influence on the next mouse. Mice were considered to be immobile when lacking any swimming movement. All animals were forced to swim for 6 min and the time spent floating was quantified as the duration of immobility for the last 4 min of the test session.

### Western blotting analysis

The mPFC were dissected rapidly from brain slices of 350 μm thickness. Brain tissue sample were immediately frozen on dry ice and stored at -80 °C. Fresh cell and frozen brain tissue were sonicated in RIPA lysis buffer containing protease and phosphatase inhibitors (50 mM Tris, 150 mM NaCl, 1% Triton X-100, 1% sodium deoxycholate, 0.1% SDS, protease inhibitor mixture, pH 7.4). Samples were then centrifuged at 12000 g for 15 min at 4 °C, and the supernatant was collected and quantified with the bicinchoninic acid assay (Beyotime Biotechnology, Haimen, China). All the protein samples were heated for 10 min at 95 °C in loading buffer. Anti-Calnexin (#ab22595, 1:500), Anti-TSG101 (#ab83, 1:500), Anti-Twist1 (#ab50887, 1:200) and Anti-PPAR-δ (#ab23673, 1:1000) were purchased from Abcam (Cambridge, MA, USA). Anti-β-actin (#sc-47778, 1:2000) was purchased from Santa Cruz Biotechnology (Waltham, MA, USA). ImageJ software (NIH, Bethesda, MD, USA) was used to quantify the optical densities of detected bands and the results were presented as the percentage of control after normalization.

### Quantitative reverse transcription-PCR

Total RNA was isolated from mPFC of mice by using TRIzol reagent (Invitrogen, Carlsbad, CA) according to the manufacturer's instructions. The full-length first strand cDNA was prepared from RNA (1 μg) by using the PrimeScript™ RT reagent Kit (TaKaRa, Dalian, China) with strand-specific primers. Template (1 µL) was amplified by real-time PCR by using 0.4 µM of each primer. Each sample was run in triplicate in a 20-µL reaction volume with the SYBR^®^ Premix Ex Taq™ II (TaKaRa, Dalian, China). The primers for real-time PCR were prepared as follows: Twist1: 5'- GAGGTCTTGCCAATCAGCCA -3' (sense), 5'- CCAGTTTGATCCCAGCGTTT -3' (anti-sense); glyceraldehyde-3-phosphate dehydrogenase (GAPDH): 5'- AACGACCCCTTCATTGAC -3' (sense), 5'- TCCACGACATACTCAGCAC -3' (antisense). Reactions were performed in 96-well plates in a Bio-Rad CFX 96 Real-Time Detection System (BIO-RAD, California, USA). PCR amplification consisted of an initial denaturation for 30 s at 94 °C, followed by 40 cycles at 95 °C for 50 s and 60 °C for 30 s. Analysis of gene expression was performed using the ΔΔCt method and normalized to housekeeping gene GAPDH.

### Analysis of dendritic morphology

For morphometric analyses of pyramidal neurons in the mPFC, single-cell microinjections were applied 17. Briefly, mice were perfused intracardially with 4% paraformaldehyde. The brains were post-fixed in paraformaldehyde at 4 °C for 12 h and coronal brain slices (300 μm thick) were cut by a VT-1000S vibratome (Leica, Wetzlar, Germany). For cell-filling injections, Lucifer yellow dye (Invitrogen L453, 100 mM in water) was loaded into a glass electrode and injected into pyramidal neurons in layer II/III of mPFC with a continuous negative 2.5 nA for 3 min until all dendritic branches appeared visible. The images were acquired on a confocal laser scanning microscope (FV1000, Olympus, Tokyo, Japan). For analysis of the dendritic structure of neurons, the lengths and branching points of dendrites were measured with the ImageJ software. Sholl analysis for dendritic complexity was carried out by counting the number of dendrites that cross a series of concentric circles at 20 μm intervals from the soma. For morphometric analysis of primary cultured cortical neurons, neurons were fixed with 4% paraformaldehyde for 2 h. The fixed neurons were then incubated with Anti-MAP2 (#ab5392, 1:200, abcam, Cambridge, UK) for 2 h, followed by Goat anti-Chicken IgY (H+L) Cross-Adsorbed Secondary Antibody, Alexa Fluor™ Plus 405 (A-48260, 1:500; ThermoFisher Scientific, USA).

### Stereotaxic injections

C57BL/6J mice were anesthetized with sodium pentobarbital (45 mg/kg, i.p.) and placed in a stereotaxic apparatus. Lentiviral shRNAs (U6-MCS-Ubi-EGFP) were used to knockdown Twist1 expression in the mPFC of mice and the target gene sequence was GCTGAGCAAGATTCAGACC. Virus used in the experiments were purchased from GeneChem company (Genechem Co., Ltd, Shanghai, China). For stereotaxic viral injections, lentiviral particles (1 µL) were bilaterally injected into the mPFC stereotaxically at the following coordinates (AP, 1.9 mm; ML, ± 0.4 mm; DV, -2.4 mm) through a Nanoliter Injector (World Precision Instruments, Sarasota). The virus was injected with a rate of 0.1 µL/min and followed by 10 min of rest to ensure optimal virus diffusion. The viral infection in brain regions was confirmed by assessment of GFP fluorescence on coronal brain slices.

### Statistics

All experimental n values represent biological replicates. Animals were randomly allocated to groups. Experimenters were blinded to the groups during the experiment and assessment. Analysis was performed by GraphPad Prism 7.0. The unpaired Student's t-tests were used for comparison of means of two independent sample groups. One-way or two-way analysis of variance followed by Dunnett's or Bonferroni's post hoc tests were used for multiple independent groups. All data were analyzed using SPSS statistical software version 18.0 (SPSS, Chicago, IL, USA) and GraphPad Prism software (ver. 7.0). Statistical significance was defined as *p* < 0.05.

## Results

### Tumor-derived EVs promotes depressive-like behaviors in tumor-bearing mice

**Figure 1:**
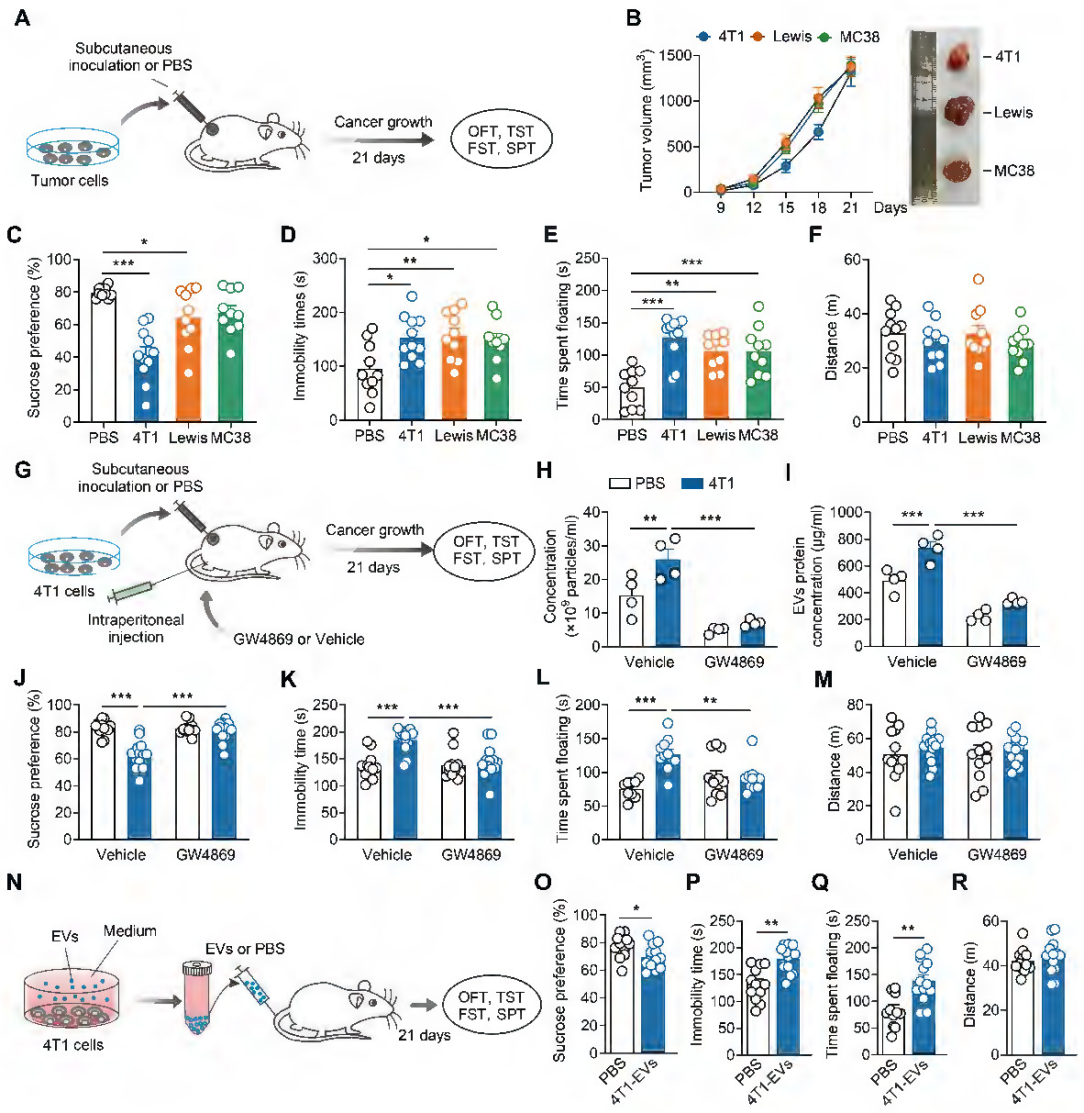
** Tumor-derived EVs promotes depressive-like behaviors. (A)** Schematic timeline of tumor cells inoculation and behavioral tests.** (B)** Average growth curves of 4T1, Lewis and MC38 tumors in female mice. Representative images of tumors at day 21 of tumor growth are shown (n = 10 mice per group). **(C-F)** Behavioral consequences of tumor cells inoculation in the SPT (**C**), TST (**D**), FST (**E**) and OFT (**F**) (n = 9-11 mice per group). **(G)** Schematic timeline of 4T1 cells inoculation, intraperitoneal injection with GW4869 and behavioral tests.** (H)** Measurement of particle number of the serum-derived EVs after treatment of GW4869 for 4T1 tumor-bearing mice (n = 4 mice per group). **(I)** Quantification of the total protein concentration in the serum-derived EVs after treatment of GW4869 for 4T1 tumor-bearing mice (n = 4 mice per group). **(J-M)** Behavioral consequences of GW4869 treatment in the SPT **(J)**, TST **(K)**, FST **(L)** and OFT **(M)** for 4T1 tumor-bearing mice (n = 9-13 mice per group). **(N)** Schematic timeline of tumor-derived EVs purification, intravenous injection and behavioral tests. **(O-R)** Behavioral consequences of intravenous injection of tumor-derived EVs in the SPT **(O)**, TST **(P)**, FST **(Q)** and OFT **(R)** (n = 12-13 mice per group). All data are presented as the mean ± s.e.m., with each point representing data from an individual. ^*^p < 0.05, ^**^p < 0.01, ^***^p < 0.001 by one-way ANOVA **(C, D, E, F)** followed by Dunnett's post hoc test or two-way ANOVA **(H, I, J, K, L, M)** followed by Bonferroni's post hoc test, or Student's *t* test **(O, P, Q, R)**. EVs, extracellular vehicles; FST, forced swimming test; OFT, open field test; PBS, phosphate buffered saline; SPT, sucrose preference test; TST, tail suspension test.

Subcutaneous tumor cell inoculation has been used to explore the biological basis of CID 28, 32. To assess depressive-like behaviors in tumor-bearing mice, we subcutaneously implanted three separate murine tumor cells (4T1 breast cancer cells, Lewis lung carcinoma cells, MC38 colon adenocarcinoma cells) into female mice, and conducted behavioral assays 21 days post-inoculation (Figure 1A). All female mice subcutaneously inoculated with tumor cells exhibited exponential tumor growth over 21 days (Figure 1B). Compared with controls, tumor-bearing mice displayed depressive-like behaviors, including anhedonia and behavioral despair, as evidenced by decreased sucrose preference in the SPT and increased immobile time in the TST and FST, respectively (Figure 1C-E). No change was found in locomotor activity in the OFT among these groups (Figure 1F). Notably, 4T1 cells-inoculated exhibited the most pronounced depressive-like phenotypes, particularly in the sucrose preference (Figure 1C). Therefore, we employed 4T1 cells to study the underlying mechanisms of CID.

Cancer cell lines have been shown to produce more EVs relative to normal (healthy) cells 33, 34. We next investigated whether tumor-derived EVs contributed to the development of CID. First, we established a standard protocol for purifying tumor-derived EVs, isolating them from the supernatant of cultured 4T1 cells via ultra-centrifugation. Transmission electron microscopy revealed that 4T1-derived EVs exhibited a typical cup-shaped morphology (Figure S1A). Protein analysis demonstrated 4T1-derived EVs expressed known surface markers of EVs, such as TSG101 and CD9 but not Calnexin, an endoplasmic reticulum marker (Figure S1B). NTA indicated that the 4T1-derived EVs were purified with featured sizes ranging from 80 to 200 nm (Figure S1C). Second, we verified the inhibitory effect of GW4869, an EVs synthesis and release inhibitor, on tumor-derived EVs. As shown in Figure S2, the ability of GW4869 to inhibit EVs secretion from the supernatant of cultured 4T1 cells was indirectly verified by a reduction in EVs surface markers as well as total secreted protein quantification. GW4869 was then intraperitoneally injected into the mice subcutaneously inoculated with 4T1 cells (Figure 1G). Subsequently, we investigated the effect of GW4869 on the generation of SDEVs *in vivo*. NTA and secreted EVs protein quantification showed that subcutaneous inoculation of 4T1 cells increased circulating EVs levels, an effect abrogated by GW4869 treatment (Figure 1H-I). As shown in Figure 1J-M, GW4869-treated mice showed no difference in the behavioral tests when compared with that of vehicle-treated controls, but prevented the depressive-like behaviors exhibited by tumor-bearing mice (Figure 1J-M), suggesting that EVs delivery is essential for the induction of depressive-like behaviors by subcutaneous 4T1 cell inoculation.

We then investigated whether tumor-derived EVs could mimic tumor-bearing and induce CID. Intranasal delivery provides a practical, noninvasive method for brain-targeted delivery, and has been widely used in EVs delivery 35. Female mice received intranasal injections of 4T1 cells-derived EVs or PBS, followed by behavioral assays 21 days post-injection (Figure S3A). The results revealed that tumor-derived EVs-treated mice exhibited decreased sucrose preference and prolonged immobility in TST and FST, while general locomotor activity remained unaffected, indicating the induction of depressive-like behaviors (Figure S3B-E). EVs can traverse the bloodstream to reach distant tissues and directly interact with target cells. To more accurately replicate the effects of EVs in tumor-bearing mice, 4T1 cells-derived EVs were subsequently administered intravenously to female mice (Figure 1N). Consistent with the intranasal administration, intravenous injection of EVs also elicited depressive-like phenotypes in female mice (Figure 1O-R). Collectively, these results support a pro-depressant role for tumor-derived EVs in tumor-bearing mice.

### Twist1/PPAR-δ signaling pathway is responsible for the depressive-like behaviors of tumor-bearing mice

**Figure 2:**
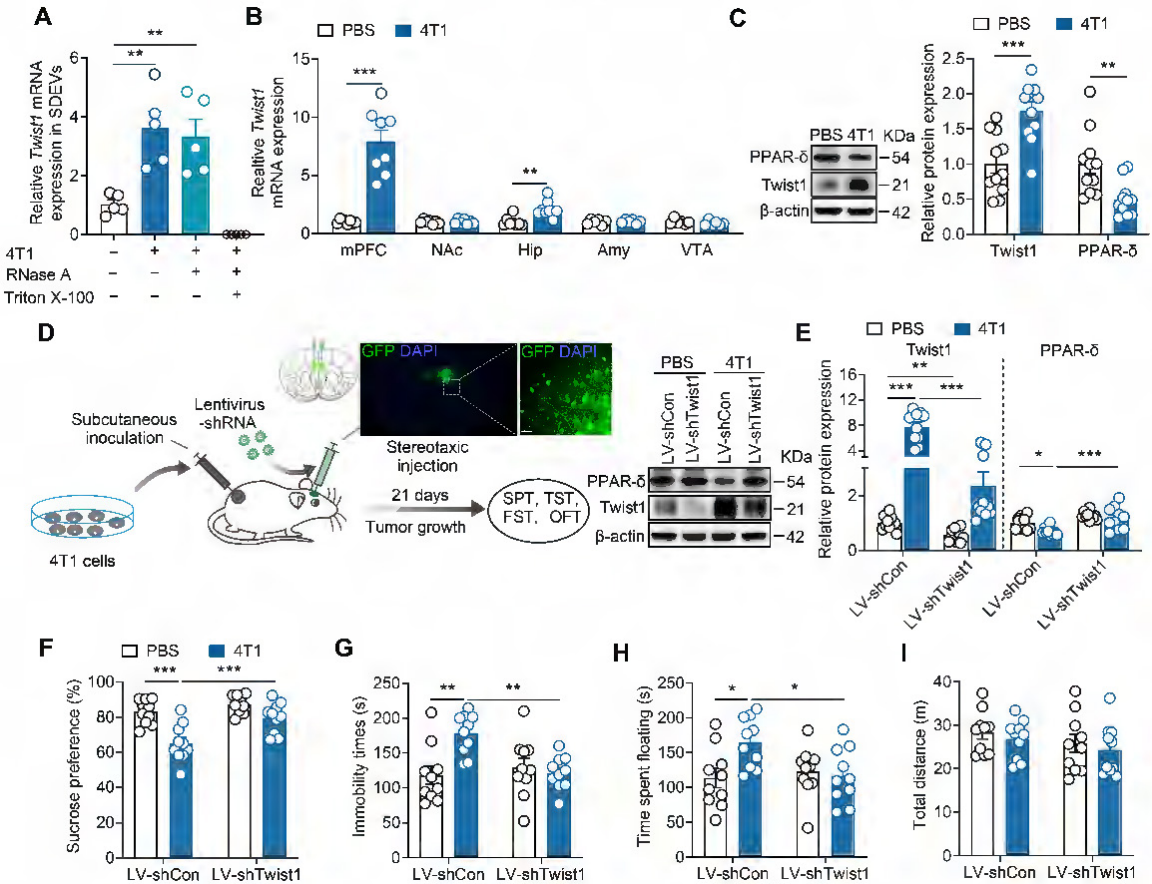
** Inhibition of Twist1/PPAR-δ signal pathway rescues depressive-like behaviors in tumor-bearing mice. (A)** Twist1 mRNA expression in the serum-derived EVs from tumor-bearing mice treated with RNase A (2 mg/mL) alone or in combination with Triton X-100 (0.1%). (n = 5 mice per group). **(B)** Twist1 mRNA expression in the mPFC, Amy, NAc, hippucampus and VTA of tumor-bearing mice (n = 6-8 mice per group). **(C)** Twist1 and PPAR-δ proteins expression in the mPFC of tumor-bearing mice (n = 11 mice per group). **(D)** Schematic timeline of 4T1 cells inoculation, lentivirus injection and behavioral tests. Targeted locations and confocal images of LV-mediated GFP (green) expression in the mPFC (right). Scale bar = 20 µm. **(E)** Twist1 and PPAR-δ proteins expression in the mPFC of tumor-bearing mice with LV-shTwist1 injection (n = 10 mice per group). **(F-I)** Behavioral consequences of viral knockdown of Twist1 in the SPT **(F)**, TST **(G)**, FST **(H)** and OFT **(I)** for tumor-bearing mice (n = 10 mice per group). All data are presented as the mean ± s.e.m., with each point representing data from an individual. ^*^p < 0.05, ^**^p < 0.01, ^***^p < 0.001 by one-way ANOVA **(A)** followed by Bonferroni's post hoc test, Student's *t* test **(B, C)** or two-way ANOVA **(E, F, G, H, I)** followed by Bonferroni's post hoc test. Amy, amygdala; FST, forced swimming test; GFP, green fluorescent protein; Hip, hippocampus; LV, lentivirus; mPFC, medial prefrontal cortex; NAc, nucleus accumbens; OFT, open field test; PBS, phosphate buffered saline; PPAR-δ, peroxisome proliferator-activated receptor-δ; SDEVs, serum-derived extracellular vehicles; SPT, sucrose preference test; TST, tail suspension test; VTA, ventral tegmental area.

Given that tumor-derived EVs package diverse molecules, including mRNAs, non-coding RNA and proteins essential for the metastatic process at target organs 36, we next identified the specific factor triggering depressive-like behaviors. Twist1, highly expressed in breast cancer, promotes tumor progression and metastasis 12, 37-39. Our previous studies found that elevated Twist1 expression correlates with depressive-like behaviors in a chronic stress model 17. Furthermore, Twist1 acts as a component of hepatocyte-derived EVs, enabling its intercellular transfer to adjacent hepatic stellate cells 40. We thus investigated whether Twist1 is necessary for the pro-depressive effect of tumor-derived EVs. EVs were purified from the serum of tumor-bearing mice followed by real-time PCR analysis. Quantitative detection revealed elevated *Twist1* mRNA in SDEVs from tumor-bearing mice (Figure 2A). To verify whether *Twist1* mRNA was encapsulated within EVs and protected from RNase A degradation, we treated SDEVs from tumor-bearing mice with RNase A (degrades free RNA) and Triton X-100 (increases cell membrane permeability). As expected, results showed no differences in *Twist1* mRNA degradation between RNase A-treated and non-treated SDEVs, whereas no mRNA was detected in samples treated with RNase A plus Triton X-100, evidencing the effective package of *Twist1* mRNA in EVs instead of being directly released (Figure 2A). To investigate how tumor-derived EVs regulate depressive-like behaviors, tumor-derived EVs were fluorescently labelled with PKH67 and intravenously injected into mice. The brain distribution of PKH67-labelled tumor-derived EVs was analyzed 24 h post-intravenous injection. It was shown that tumor-derived EVs were widely distributed in the brain, including mPFC, nucleus accumbens, hippocampus and amygdala, suggesting the ability of tumor-derived EVs to target the brain after being released into the peripheral blood (Figure S4). Among these regions, *Twist1* mRNA levels were upregulated in the mPFC and hippocampus of tumor-bearing mice (Figure 2B). Moreover, western blot analysis confirmed higher levels of Twist1 protein in the mPFC (Figure 2C). Chronic stress-inducible Twist1 promotes depressive-like behaviors through inhibition of PPAR-δ in the mPFC 17. We also observed lower expression of PPAR-δ protein in the mPFC of tumor-bearing mice, indicating activation of Twist1/PPAR-δ signaling pathway.

To evaluate the contributions of inoculation of tumor cells-induced Twist1 in the mPFC to CID, Twist1 was knocked down via intra-mPFC injection of lentivirus (LV) harboring short hairpin RNA targets *Twist1* (LV-shTwist1) (Figure 2D). We observed that knockdown of Twist1 restored PPAR-δ expression in the mPFC of tumor-bearing mice (Figure 2E). More importantly, knockdown of Twist1 in the mPFC prevented of 4T1 cells-inoculation-induced depressive-like behaviors, including anhedonia in the SPT, and behavioral despair in the TST and FST (Figure 2F-I). These results suggest that Twist1/PPAR-δ signaling pathway in the mPFC contributes to CID.

### Genetic knockdown of Twist1 in tumor cells prevents depressive-like behaviors in tumor-bearing mice

**Figure 3:**
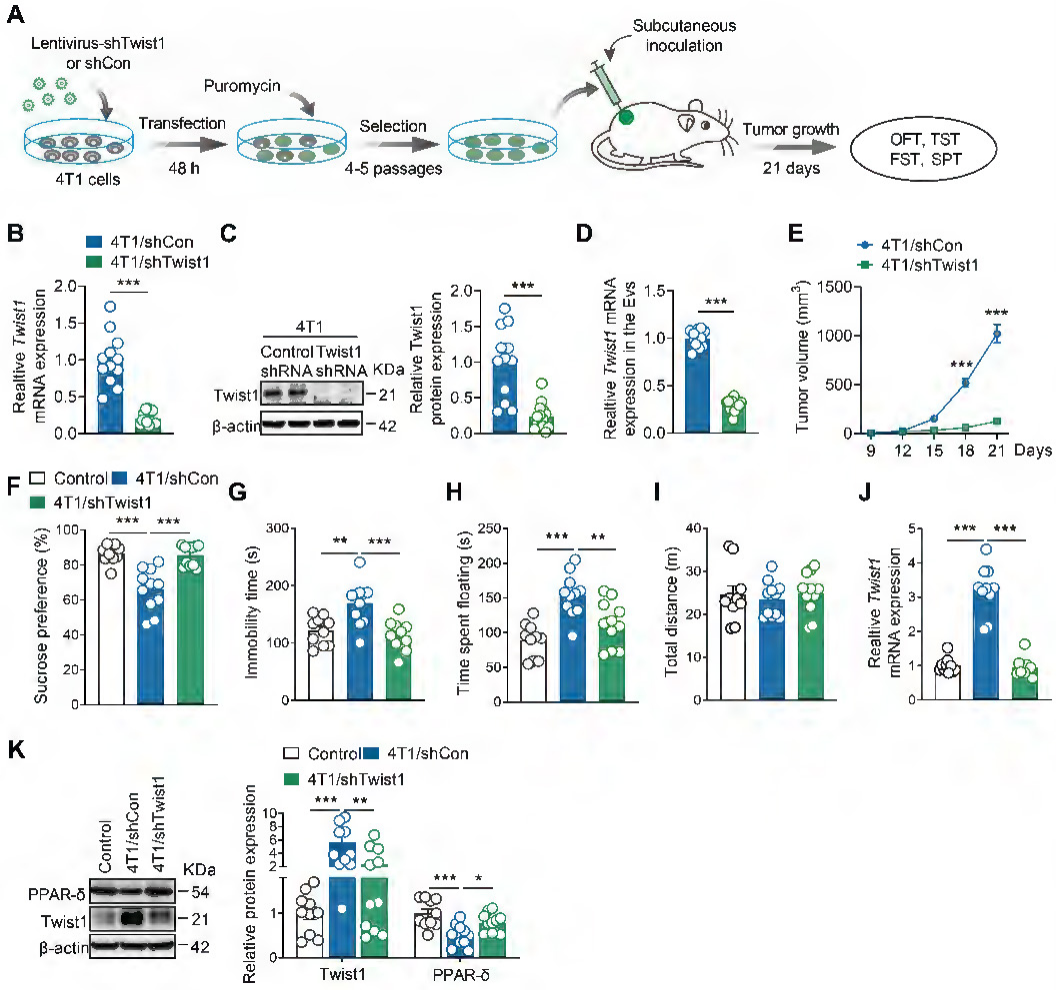
** Genetic knockdown of Twist1 in tumor cells prevents depressive-like behaviors in tumor-bearing mice. (A)** Schematic timeline of stable 4T1 cells generation and inoculation and behavioral tests. **(B)** Twist1 mRNA expression of 4T1 cells after lentiviral infection and puromycin selection (n = 12-13 cultures per group). **(C)** Twist1 protein expression of 4T1 cells after lentiviral infection and puromycin selection (n = 12-14 cultures per group). **(D)** Twist1 mRNA expression of 4T1 cells-derived EVs after lentiviral infection and puromycin selection (n = 12 cultures per group). **(E)** Average growth curves of tumor in mice subcutaneously inoculated with stable 4T1 cell lines expressing Twist1 shRNA or control shRNA (n = 10 mice per group). **(F-I)** Behavioral consequences of subcutaneous inoculation of stable 4T1 cell lines expressing Twist1 shRNA in the SPT **(F)**, TST **(G)**, FST **(H)** and OFT** (I)** (n = 10-11 mice per group). **(J)** Twist1 mRNA expression in the mPFC of mice subcutaneously inoculated with stable 4T1 cell lines expressing Twist1 shRNA or control shRNA (n = 10-11 mice per group). **(K)** Twist1 and PPAR-δ proteins expression in the mPFC of mice subcutaneously inoculated with stable 4T1 cell lines expressing Twist1 shRNA or control shRNA (n = 10-11 mice per group). All data are presented as the mean ± s.e.m., with each point representing data from an individual. ^*^p < 0.05, ^**^p < 0.01, ^***^p < 0.001 by Student's *t* test **(B, C, D, E)** or one-way ANOVA **(F, G, H, I, J, K)** followed by Bonferroni's post hoc test. FST, forced swimming test; OFT, open field test; PPAR-δ, peroxisome proliferator-activated receptor-δ; SPT, sucrose preference test; TST, tail suspension test.

Next, we aimed to identify whether tumor cells-derived Twist1 is essential for CID. We established stable 4T1 cell lines expressing Twist1 shRNA via lentiviral infection and puromycin selection (Figure 3A). Real-time PCR and western blot analyses confirmed a significant reduction in Twist1 mRNA and protein expression in stable 4T1 cell lines (Figure 3B-C). Meanwhile, it was found that Twist1 mRNA levels in tumor-derived EVs were reduced in Twist1 shRNA-expressing 4T1 cells compared to control shRNA-expressing cells (Figure 3D). Stable Twist1 shRNA-expressing 4T1 cells were then subcutaneously implanted in female mice, followed by behavioral tests 21 days later (Figure 3A). Consistent with previous studies 41, 42, Twist1 suppression inhibited tumor growth (Figure 3E). Further behavioral tests demonstrated that knockdown of tumor cells-derived Twist1 abolished depressive-like behaviors in tumor-bearing mice (Figure 3F-J). Meanwhile, it was accompanied by reduced Twist1 and increased PPAR-δ expression in the mPFC (Figure 3K). Overall, our findings support the association of tumor cells-derived Twist1 with elevated Twist1 in the mPFC of tumor-bearing mice, suggesting the pro-depressant effects of tumor cells-derived Twist1.

### EVs-packaged Twist1 from serum of tumor-bearing mice mediates the depressive-like behaviors

**Figure 4:**
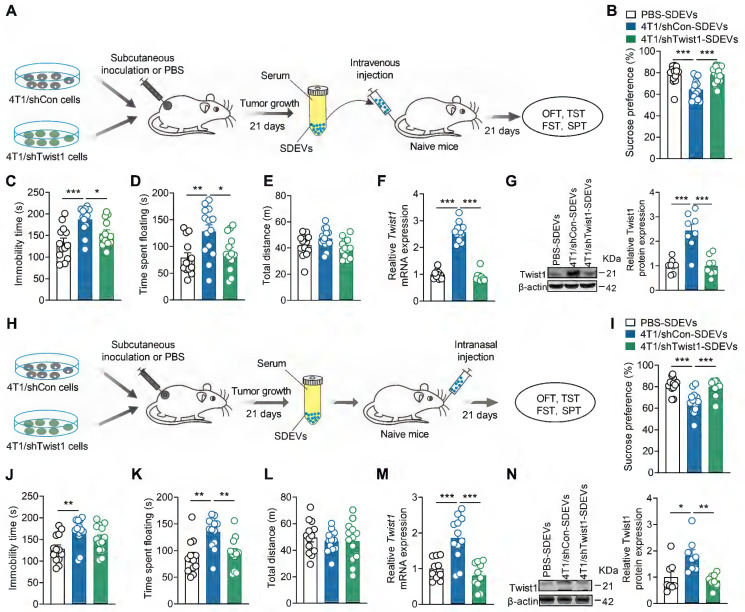
** Administration of EVs-packaged Twist1 from serum of tumor-bearing mice induces depressive-like behaviors in naïve mice. (A)** Schematic timeline of 4T1 cells inoculation, SDEVs purification, intravenous injection and behavioral tests. **(B-E)** Behavioral consequences of intravenous injection of SDEVs from tumor-bearing mice in the SPT **(B)**, TST **(C)**, FST **(D)** and OFT **(E)** (n = 13-14 mice per group). **(F, G)** Twist1 mRNA **(F)** and protein **(G)** expression in the mPFC of mice with intravenous injection of SDEVs from tumor-bearing mice (n = 8-12 mice per group). **(H)** Schematic timeline of 4T1 cells inoculation, SDEVs purification, intranasal injection and behavioral tests. **(I-L)** Behavioral consequences of intravenous injection of SDEVs from tumor-bearing mice in the SPT (**I**), TST **(J)**, FST **(K)** and OFT **(L)** (n = 12-13 mice per group). **(M, N)** Twist1 mRNA **(M)** and protein **(N)** expression in the mPFC of mice with intranasal injection of SDEVs from tumor-bearing mice (n = 8-12 mice per group). All data are presented as the mean ± s.e.m., with each point representing data from an individual. ^*^p < 0.05, ^**^p < 0.01, ^***^p < 0.001 by one-way ANOVA followed by Bonferroni's post hoc test. FST, forced swimming test; OFT, open field test; PBS, phosphate buffered saline; SDEVs, serum-derived extracellular vehicles; SPT, sucrose preference test; TST, tail suspension test.

Given that tumor-derived EVs promotes CID, we questioned whether tumor-bearing mice exhibit depressive-like behaviors by releasing EV_S_ into the blood and target the mPFC. To elucidate this, SDEVs were isolated from tumor-bearing mice and intravenously injected into naïve mice (Figure 4A). We found that the mice treated with SDEVs from 4T1 cell tumor-bearing mice showed depressive-like behaviors in the SPT (Figure 4B), TST (Figure 4C) and FST (Figure 4D), suggesting that SDEVs contribute to CID pathogenesis. SDEVs treatment did not change locomotion of mice in the OFT (Figure 4E), suggesting that the pro-depressant effects of SDEs are independent of locomotor changes. To further investigate whether behavioral effects of SDEVs from tumor-bearing mice was associated with tumor cell-derived Twist1, stable 4T1 cell lines expressing Twist1 shRNA were applied. Results showed that SDEVs from stable 4T1 cell tumor-bearing mice failed to induce behavioral abnormalities (Figure 4B-E). In addition, intravenous injection of SDEVs from 4T1 cell tumor-bearing mice increased mRNA and protein expression of Twist1 in the mPFC, which was suppressed when treatment with SDEVs from stable 4T1 cell tumor-bearing mice (Figure 4F-G). These results suggest that SDEVs from 4T1 tumor-bearing mice promote Twist1 expression in recipient cells via EVs-mediated transfer.

Compared to intravenous administration, intranasal delivery has emerged as a more effective route for delivering cargo to the brain while bypassing the blood-brain barrier 43. To further validate the role of serum-derived EVs-packaged Twist1 from tumor-bearing mice in CID, we replicated the above experiment via intranasal administration (Figure 4H). Consistent with our prior findings, knockdown of tumor cell-derived Twist1 prevented the pro-depressant effects of intravenous administration of SDEVs from 4T1 cell tumor-bearing mice at both behavioral and biochemical levels (Figure 4I-N). These findings demonstrate that EVs-mediated Twsit1 from remote tumor cells reach the brain via peripheral blood pathways to induce depressive-like behaviors.

### EVs-packaged Twist1 induces defective neuronal morphogenesis of dendrites

**Figure 5:**
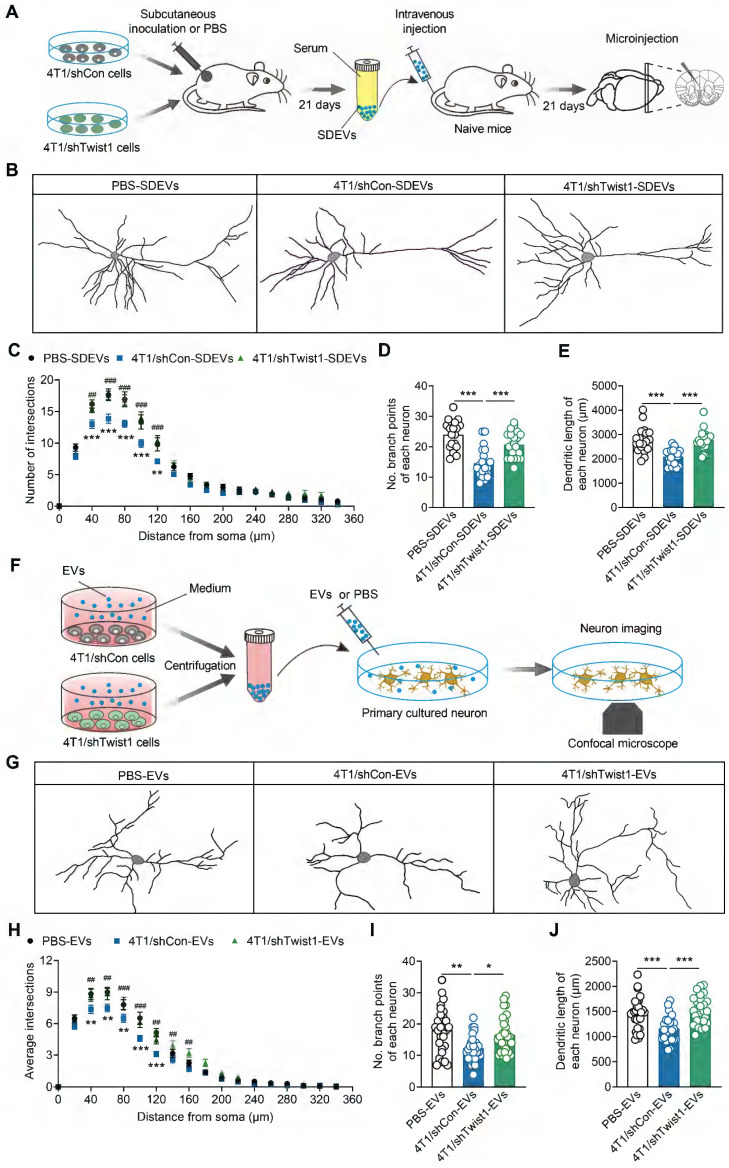
** Tumor derived-EVs-packaged Twist1 induces defective neuronal dendritogenesis. (A)** Schematic timeline of 4T1 cells inoculation, SDEVs purification, intravenous injection and microinjection. **(B)** Representative image of 3-dimensional reconstruction of dendrites by infusion of lucifer yellow into mPFC layer II/III pyramidal neurons of mice with intravenous injection of SDEVs from tumor-bearing mice. **(C-E)** Sholl analysis **(C)** and quantification of branch number **(D)** and total dendritic length **(E)** of dendrites from mPFC layer II/III pyramidal neurons of mice with intravenous injection of SDEVs from tumor-bearing mice (n = 20 neurons per group). In **(C)**: ^**^p < 0.01, ^***^p < 0.001 for PBS-SDEVs vs. 4T1/shCon-SDEVs; ^##^*p* < 0.01, ^###^*p* < 0.001 for 4T1/shCon-SDEVs vs. 4T1/shTwist1-SDEVs. **(F)** Schematic timeline of tumor-derived EVs purification, treatment and neuron imaging. **(G)** Representative image of 3-dimensional reconstruction of dendrites from primary cultured neurons with tumor-derived EVs treatment. **(H-J)** Sholl analysis **(H)** and quantification of branch number **(I)** and total dendritic length **(J)** of dendrites from primary cultured neurons with tumor-derived EVs treatment (n = 29 neurons per group). In **(H)**: ^**^p < 0.01, ^***^p < 0.001 for PBS-EVs vs. 4T1/shCon-EVs; ^##^*p* < 0.01, ^###^*p* < 0.001 for 4T1/shCon-EVs vs. 4T1/shTwist1-EVs.All data are presented as the mean ± s.e.m., with each point representing data from an individual. ^*^p < 0.05, ^**^p < 0.01, ^***^p < 0.001 by one-way ANOVA followed by Bonferroni's post hoc test. PBS, phosphate buffered saline; SDEVs, serum-derived extracellular vehicles.

Previous studies have demonstrated that increased Twist1 expression promotes dendritic atrophy in the mPFC, which is associated with depression 17, 44, 45. Therefore, we investigated whether tumor cell-derived EVs-encapsulated Twist1 regulates dendritic morphogenesis *in vivo*. SDEVs from 4T1 cells expressing control shRNA or Twist1 shRNA cell tumor-bearing mice were intravenously injected into recipient mice, followed by microinjection with lucifer yellow into pyramidal neurons from layer II/III of the mPFC (Figure 5A). We found reduced dendrite complexity, branch number and dendritic length in recipient mice injected with SDEVs from 4T1 cells tumor-bearing mice (Figure 5B-E). Conversely, knockdown of Twist1 in tumor cells blocked SDEVs-elicited aberrant dendritic structures (Figure 5B-E).

To investigate the direct effects of tumor-derived EVs on neurons, an *in vitro* experiment was conducted by co-culturing primary cortical neurons with low-Twist1 EVs (Figure 5F). As shown in Figure 5G, primary cortical neurons co-cultured with 4T1 cell tumor-derived EVs displayed dendritic atrophy phenotype, characterized by reduced dendrite complexity and decreased branch number and dendritic length, an effect rescued by knockdown of Twist1 in stable 4T1 cells (Figure 5G-J). These results suggest that the tumor cell-derived EVs-packaged Twist1, at least in part, induces defective neuronal dendrite morphogenesis. Collectively, these findings confirm that tumor cell-derived EVs-packaged Twist1 promotes neuronal dendritic atrophy phenotype.

## Discussion

**Figure 6:**
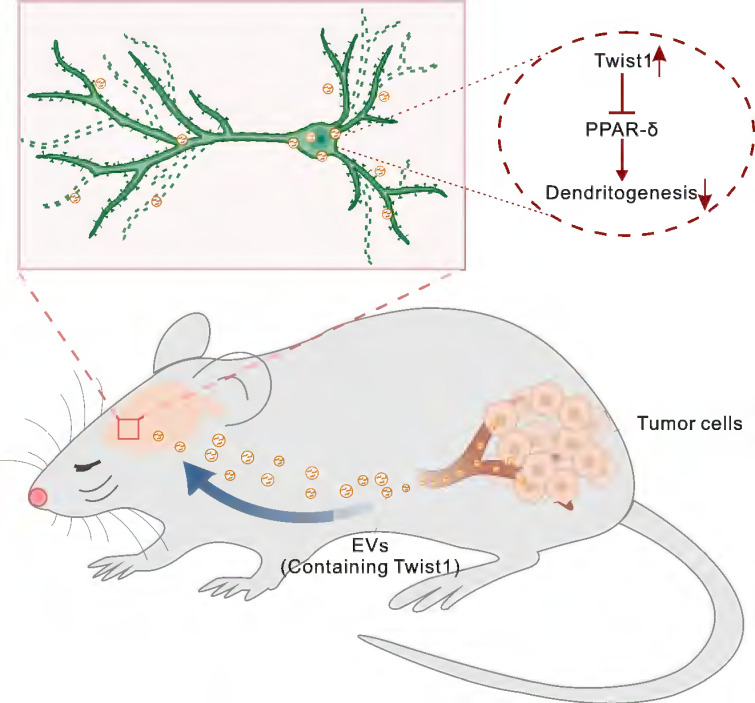
** Schematic representation of the suggested model for the role of tumor derived-EVs-packaged Twist1 in depressive-like behaviors.** Primary tumor derived-EVs-packaged Twist1 was released into the circulation and accumulates in the mPFC, which leads to defective neuronal dendritogenesis and mediates depressive-like behaviors in tumor-bearing mice. EVs, extracellular vehicles; PPAR-δ, peroxisome proliferator-activated receptor-δ.

Gaining insight into the mechanisms of CID and the specific contribution of tumor-derived EVs to this process provides opportunities for treatment of CID. In the present study, we proposed an EVs-mediated communication mode between brain and distant tumor. We demonstrated that EVs-packaged Twist1 derived from tumor cells induced dendritic atrophy and elicited depressive-like behaviors (Figure 6). While cellular expression of Twist1 has been associated with cancer invasion and metastasis, our study uncovers a novel finding: 4T1 cell-derived EVs-packaged Twist1, targeting the mPFC, plays a pivotal role as a key crucial contributor to CID.

Cancer patients with comorbid depression exhibit poorer quality of life and lower treatment-seeking/compliance rates. The application of typical antidepressants in cancer therapy remains highly controversial. In animal models, fluoxetine administration post-melanoma cell injection inhibited tumor growth, whereas chronic pre-treatment with fluoxetine before melanoma bearing accelerated metastasis via impairment of protective immune responses 46, 47. Although serotonin reuptake inhibitors (SSRIs) have proven effective in alleviating anxiety and depression among cancer patients 48, prolonged usage of SSRIs has been linked to a heightened risk of developing breast and ovarian cancers 49. The crux of the matter behind these inconsistent research findings lies in the failure to clearly elucidate the precise mechanism that connects the distinctive characteristics of cancer biology to the onset of depression. Subcutaneous inoculation of tumor cells has been employed to explore the underlying mechanisms of cancer-associated complications, such as metastasis, anorexia, and fatigue 50-52. Tumor-bearing mouse models, particularly those subcutaneous implantation of 4T1 breast cancer cells, are widely used for studying CID 28, 32, 53, 54. We implanted three separate murine tumor cells (4T1, Lewis, MC38) to establish mouse models of breast cancer, lung cancer and colon cancer. Although all three models induced depressive-like behaviors, 4T1 cells tumor-bearing mice exhibited more pronounced anhedonia, consistent with the higher prevalence of depressive disorders among breast cancer patients 55, 56. Our work has shown that 4T cell-derived EVs-packaged Twist1 induces defective neuronal morphogenesis in the mPFC, providing mechanistic insights into tumor-derived EVs-mediated CID. Further dissection and functional verification of EVs cargo from various cancers will be critical to determine whether this mechanism is applicable to other types of CID. It is worth noting that not every instance of CID can be attributed to the EVs-packaged Twist1 pathway, given that cancer and depression converge on several key pathophysio-logical mechanisms, including inflammation and aberrant neurotransmission 57.

Brain metastases affect approximately 20%-40% of patients with malignancies. Lung cancers are the most common source of brain metastases (40%-50%), followed by breast cancer (15%-20%) 58. Furthermore, EVs play a pivotal role in driving the progression of cancer metastasis. Uptake of brain EVs establishes a pre-metastatic niche for tumor localization and metastasis 59, 60. Mammary inoculation of 4T1 cells serves as a preclinical models of brain metastases in breast cancer, with micro-metastatic 4T1 cells detectable in the brain 61, 62. Although whether subcutaneous inoculation of 4T1 cells induces brain metastasis remains unclear, circulating tumor cells may reach the mPFC, change the local microenvironment, and upregulate Twist1 expression to induce neuronal dendritic atrophy in tumor-bearing mice. Tumor-derived EVs may also remodel the local microenvironment of the mPFC, thereby activating Twist1/PPAR-δ signaling pathway.

In this study, we established a stable 4T1 cell line expressing Twist1 shRNA and confirmed a significant reduction in tumor cell-derived EVs-packaged Twist1. We conclude that Twsit1 expression in the mPFC induced by tumor-derived EVs is dependent on EVs-mediated Twist1 transfer. Several lines of evidence support this conclusion: (1) *In vivo* analysis demonstrated that subcutaneous inoculation of 4T1 cells or administration with SDEVs from tumor-bearing mice increased the expression of Twist1 in the mPFC; (2) Elimination of Twist1 from 4T1 cell decreased Twist1 mRNA and protein levels in the mPFC of recipient mice; (3) Inhibiting EVs synthesis by intraperitoneal injection of GW4869 improves depressive-like behavior in tumor-bearing mice. EVs can package various bioactive cargos, including RNA, protein, lipids and metabolites to exert systemic regulation 18. In our model, knockdown of Twist1 by intra-mPFC injection of lentivirus prevents depressive-like behavior in tumor-bearing mice, suggesting that tumor-derived EVs are more likely to carry Twist1 mRNA. EVs confer protection against RNase degradation and enhance the stability of packaged RNA. RNase treatment has been used for identification of EVs-derived RNA 63. RNase digestion alone failed to alter *Twist1* mRNA expression levels in SDEVs from tumor-bearing mice, whereas combined treatment with Triton X-100 substantially decreased *Twist1* mRNA abundance. Administration of SDEVs from tumor-bearing mice increased the Twist1 mRNA expression in the mPFC, which could be rescued by knockdown of Twist1 in the 4T1 cells. These data indicate that the increase in Twist1 mRNA in the mPFC of tumor-bearing mice is attributed to EVs-packaged Twist1 mRNA.

Structural brain alterations following cancer and its treatment were reported in a majority of the publications as evidenced by reduced global/local gray matter volumes, impaired white matter microstructural integrity, and altered brain network 64. A structural magnetic resonance imaging (MRI) study showed that non-small-cell lung cancer patients exhibit a widespread white matter damage compared with healthy controls group 65. Breast cancer patients show significantly reduced activation in the left middle dorsolateral prefrontal cortex in the functional MRI test, and reduced activation was significantly correlated with higher disease severity 66. Another case study involving a breast cancer patient comorbid with depression demonstrated increased PFC activity following an 8-session behavioral activation therapy for depression 67. Although the baseline (pre-systemic therapy) research on brain structure and function is relatively lacking, these findings suggest that cancer patients are at higher risk for brain structure and function alterations. However, the previous studies primarily focused on global brain structural and functional changes. Cellular-level structural changes in the brains of cancer patients and their association with CID remain unclear. Clinical and post-mortem studies using neuroimaging and volumetric analyses have consistently identified dendritic atrophy and reduced brain activation in the mPFC of depressed patients 68-70. In our study, we demonstrated that tumor-bearing mice exhibited depressive-like behaviors accompanied by neuronal dendritic atrophy, consistent with previous studies 28. More importantly, we identified the key role of tumor cell-derived EVs-packaged Twist1 in neuronal morphogenesis regulation. It is worth noting that tumor-derived exosomes also induce tumor-localized axonogenesis and innervation, a process potentiated by the exosome-packaged EphrinB1, an axonal guidance molecule 71. Future post-mortem studies in cancer patients will help verify whether Twist1 expression changes in the mPFC, and accompanied by abnormal neuronal morphogenesis.

The most promising therapeutic implication of our findings lies in the observation that inhibition of EVs secretion from tumor cells elicits antidepressant effects in cancer patients. Here, we employed a pharmacological approach to dissect the role of tumor-derived EVs in CID by treatment with GW4869. GW4869, a blocker of neutral sphingomyelinase-2 (nSMase2), mediates the biogenesis and secretion of both tumor-derived EVs and non-tumor-derived EVs via an endosomal sorting complex required for transport-independent way 72. Thus, direct application of GW4896 without tumor-targeted delivery may lead to nonspecific inhibition of both tumor-derived and non-tumor-derived EVs 73. Biosafety assessment of GW4869 is required before its clinical application, as nSMase2 regulates multiple central biological processes, potentially causing side effects *in vivo* 74. Although tumor-derived and non-tumor-derived EVs share some underlying mechanisms, their biogenesis and secretion processes exhibit notable differences, primarily attributed to the aberrant expression of regulatory factors in tumor-derived EVs. Genomic mutations underlying aberrant biogenesis and secretion of tumor-derived EVs may serve as specific therapeutic targets 75. For example, as a potent regulator of EVs secretion, ablation of Rab27a suppresses EVs secretion by tumor cells 50, 76. More effective small-molecule inhibitors targeting these mechanisms are warranted. Strategies to reduce tumor-derived Twist1 should also be explored, as they could both prevent CID and inhibit tumor growth. It would be interesting to investigate whether localized anti-EVs therapy combined with systemic anti-Twist1 blockade could synergize to exert therapeutic effects on cancer progression and CID.

## Conclusion

Overall, this study reveals that EVs-packaged Twist1 is a key driver of CID. Tumor cell-derived Twist1 is transported via EVs in the bloodstream to the mPFC, where it subsequently induces dendritic atrophy and depressive-like behaviors in mice. Intravenous/intranasal tumor-derived EVs administration recapitulates CID phenotypes. Genetic knockdown of Twist1 in tumor cells or pharmacological inhibition of EVs secretion (GW4869) reverses these effects. These findings establish the tumor-derived EVs or their cargo Twist1 as a novel target, offering potential strategies like tumor-specific EVs blockade for CID treatment.

### Limitations of the study

Throughout this study, the increased Twist1 expression in the mPFC of tumor-bearing mice was primarily attributed to tumor-derived EVs. However, we cannot rule out the possibility of endogenous *Twist1* mRNA production by neurons, which may involve stimulation by tumor-derived EVs or potential stress induced by subcutaneous tumor growth. Additionally, the precise mechanism linking Twist1 expression to neuronal dendritic atrophy in the mPFC of tumor-bearing mice remains undefined. Finally, the present study did not examine Twist1 expression changes in SDEVs from clinical cancer patients with comorbid depression. Whether administration of such patient-derived SDEVs into naïve mice can induce depressive phenotypes and neuronal morphological alterations warrants further investigation.

## Supplementary Material

Supplementary figures and tables.

## Abbreviations

CID: cancer-induced depression
EVs: extracellular vehicles
FST: forced swim test
GFP: green fluorescent protein
LV: lentivirus
mPFC: medial prefrontal cortex
NAc: nucleus accumbens
nSMase2: neutral sphingomyelinase-2
NTA: nanoparticle tracking analysis
OFT: open field test
PBS: phosphate-buffered saline
PPAR-δ: peroxisome proliferator-activated receptor-δ
SDEVs: serum-derived extracellular vehicles
SPT: sucrose preference test
SSRIs: serotonin reuptake inhibitors
TST: tail suspension test
