# Supplementary material for: Extracellular vehicles-mediated Twsit1 transferred from tumor cells to brain induces depressive-like behaviors via neuronal morphogenesis
Source: Theranostics. 2025 Aug 16;15(17):8985–9000. doi: 10.7150/thno.112238 (PMC12439268; doi:10.7150/thno.112238)
Supplement: Supplementary file 1 — Supplementary figures and tables. [file thnov15p8985s1.pdf]

Supplementary Figures

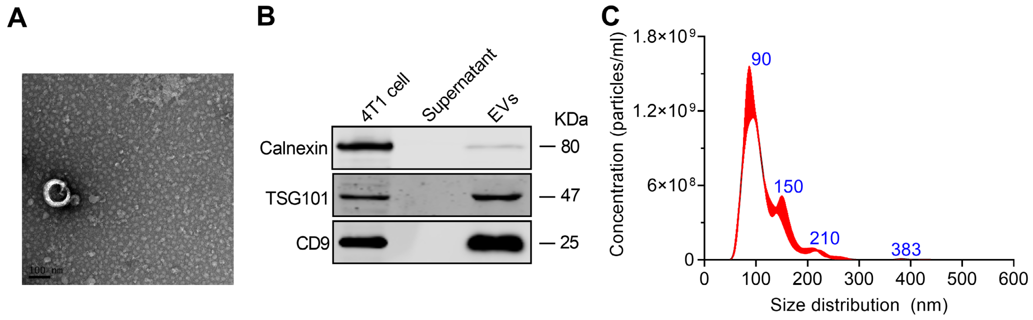

Figure S1. Characteristics of 4T1 cells-derived EVs. (A) Representative electron microscopy images of 4T1 cells-derived EVs. Scale bar = 100 nm. **(B)** Western blot analysis of the EVs markers TSG101, CD9 of 4T1 cells-derived exosomes. **(C)** Measurement of particle size of the 4T1 cells-derived EVs by nanoparticle tracking analysis. EVs, extracellular vehicles.

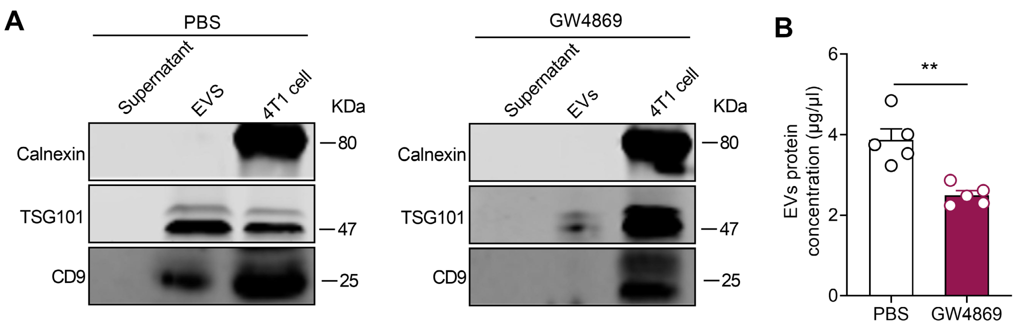

Figure S2. GW4869 inhibits EVs secretion in 4T1 cells. (A) Western blot analysis of the expression of exosomal markers TSG101 and CD9 in 4T1 cells-derived EVs after treatment of GW4869. **(B)** Quantification of the total protein concentration in the 4T1 cells-derived EVs after treatment of GW4869. All data are presented as the mean ± s.e.m., with each point representing data from an individual. $^{**}p < 0.01$ by Student's t test. EVs, extracellular vehicles; PBS, phosphate buffered saline .

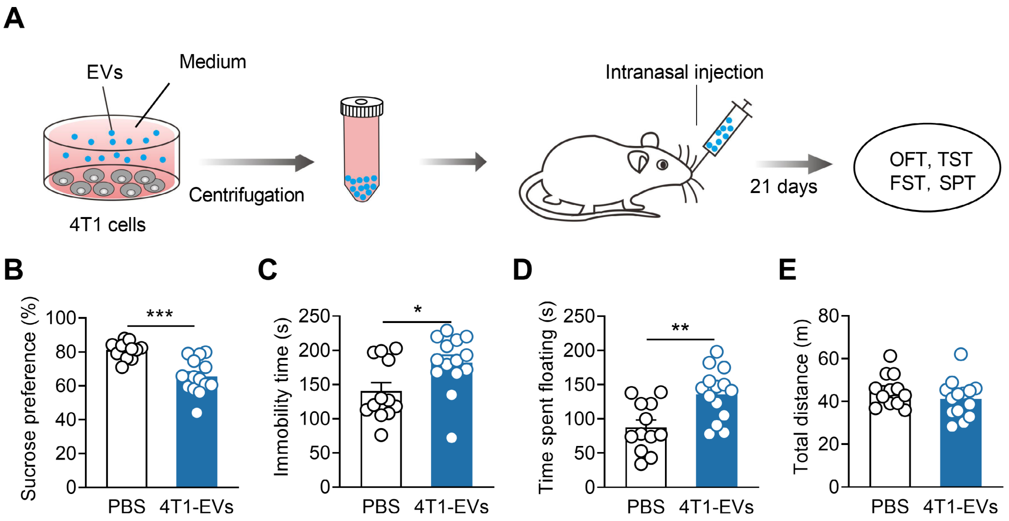

Figure S3. Intranasal administration of 4T1 cells-derived EVs induces depressive-like behaviors in mice. (A) Schematic timeline of 4T1 cells-derived EVs purification, intranasal injection and behavioral tests. **(B-E)** Behavioral consequences of intranasal injection of 4T1 cells-derived EVs in the SPT **(B)**, TST **(C)**, FST **(D)** and OFT **(E)** (n = 12-14 mice per group). All data are presented as the mean ± s.e.m., with each point representing data from an individual. *$p < 0.05$, **$p < 0.01$, ***$p < 0.001$ by Student's t test. EVs, extracellular vehicles; FST, forced swimming test; OFT, open field test; PBS, phosphate buffered saline; SPT, sucrose preference test; TST, tail suspension test.

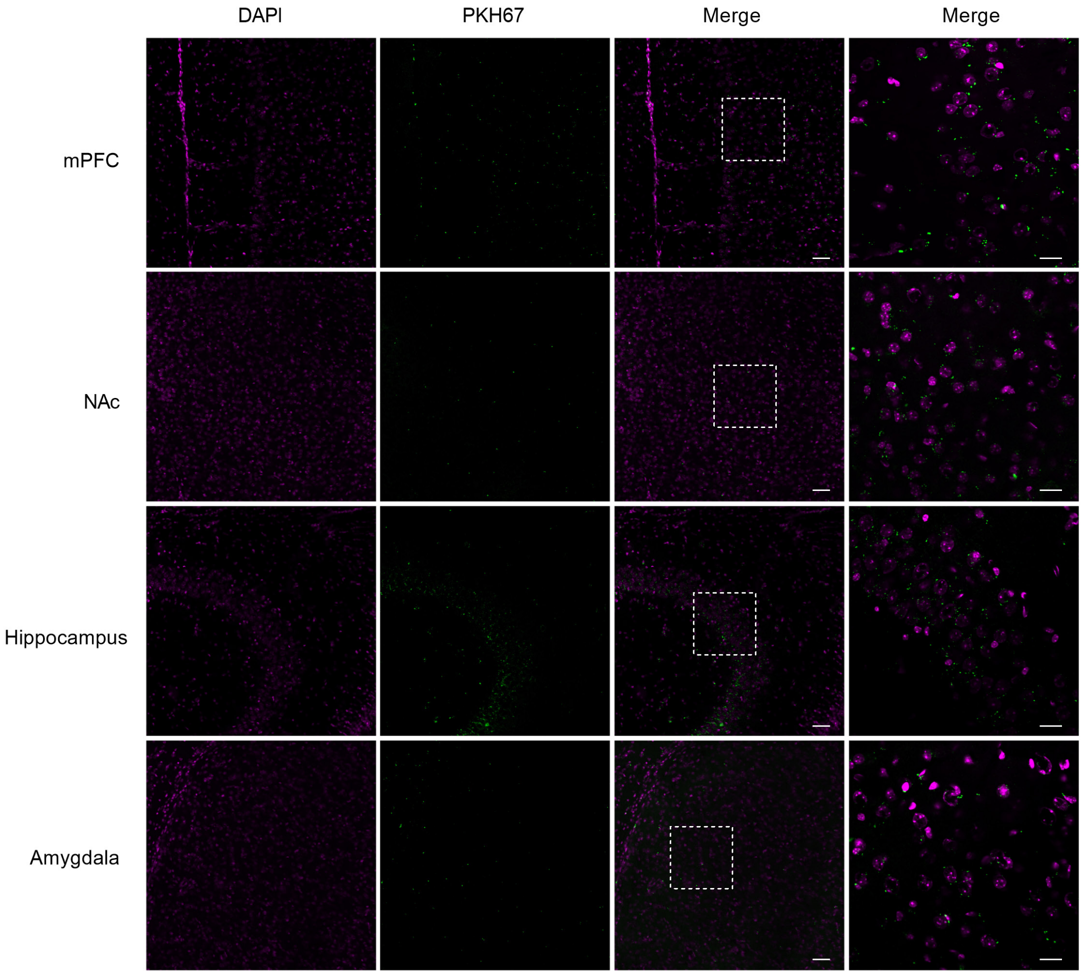

Figure S4. Distribution of intravenously injected 4T1 cells-derived EVs in the brain. Higher magnification views of selected regions (white rectangle). Scale bar = 50 μm (left), 20 μm (right). mPFC, medial prefrontal cortex; NAc, nucleus accumbens.

Supplementary Table 1. Key reagent or resource.

**Reagent or resource**	**Source**	**Identifier**
**Antibodies**		
4',6-diamidino-2-phenylindole	Sigma	D9542
Alexa Fluor™ Plus 405	ThermoFisher Scientific	A-48260; RRID: AB_2890271
Chicken-anti-MAP2	Abcam	ab5392; RRID: AB_2138153
IRDye 800CW Goat anti-Mouse IgG	LI-COR	926-32210; RRID: AB_621842
IRDye 800CW Goat anti-Rabbit IgG	LI-COR	926-32211; RRID: AB_621843
Mouse-anti-TSG101	Abcam	ab83; RRID: AB_306450
Mouse-anti-Twist1	Abcam	ab50887; RRID: AB_883294
Mouse-anti-β-actin	Santa Cruz Biotechnology	sc-47778; RRID: AB_626632
PKH67	NoninBio	NW3217
Rabbit-anti-Calnexin	Abcam	ab22595; RRID: AB_2069006
Rabbit-anti-PPAR-δ	Abcam	ab23673; RRID: AB_2165902
**Virus strains**		
LV-Twist-RNAi (3×109 TU/mL)	Genechem	Cat#133C77A
**Chemicals**		
B-27 Supplement (50X)	Gibco	Cat#17504-044
Deoxyribonuclease I	Merck	Cat#DN25
Dulbecco's Modified Eagle Medium	Gibco	Cat#C11330500BT
Fetal Bovine Serum	Gibco	Cat#10099141C
Glutamax	Gibco	Cat#25030081
GW4869	MCE	Cat#HY-19363
Lucifer yellow dye	Invitrogen	Cat# L453
Neurobasal medium	Gibco	Cat#21103049
Puromycin	Solarbio	Cat#P3280
RIPA Lysis Buffer	Beyotime	Cat#P0013C
RNAiso Blood	Takara	Cat#9112
RNase A	Takara	Cat#2158
SYBR® Premix Ex Taq™ II	Takara	Cat#RR820A
Triton X-100	Biofroxx	Cat#1139ML100
**Critical commercial assays**		
Bicinchoninic acid assay	Beyotime	Cat#P0010
PrimeScript™ RT reagent Kit	Takara	Cat#RR037Q

**Oligonucleotides**		
Twist1: 5'-GAGGTCTTGCCAATCAGCCA -3' (sense)	Invitrogen	N/A
Twist1:5'-CCAGTTTGATCCCAGCGTTT -3' (antisense)	Invitrogen	N/A
GAPDH: 5'-AACGACCCCTTCATTGAC -3' (sense)	Invitrogen	N/A
GAPDH: 5'-TCCACGACATACTCAGCAC -3' (antisense)	Invitrogen	N/A
**Software and algotithms**		
NF Profession 2.0 software	Nacofcm	https://www.nanofcm.cn/
ANY-maze software	Stoelting Co.	https://stoeltingco.com/
ImageJ	National Institute of Health	https://imagej.net/ij/
SPSS statistical software version 18.0	IBM	https://www.ibm.com/cn-zh/products/spss-statistics
GraphPad Prism software (ver. 7.0)	GraphPad	https://www.graphpad.com/scientific-software/prism/

Supplementary Table 2. Statistical analysis for Figures 1-5 and Supplementary Figure 1-3.

Figure and numbers	Statistical analysis	*Post hoc tests*	Means ± s.e.m.
**1B**	**Two-way ANOVA**	**Bonferroni's multiple comparisons test**	
4T1 = 10<br>Lewis = 10<br>MC38 = 10	Days: F (4, 133) = 155.2, P < 0.0001<br>Tumor: F (2, 133) = 5.751, P = 0.004<br>Days × Tumor = F (8, 133) = 1.128, P = 0.3489	**9:**<br>4T1 vs. Lewis, P > 0.9999<br>4T1 vs. MC38, P > 0.9999<br>Lewis vs. MC38, P > 0.9999<br>**12:**<br>4T1 vs. Lewis, P > 0.9999<br>4T1 vs. MC38, P > 0.9999<br>Lewis vs. MC38, P > 0.9999<br>**15:**<br>4T1 vs. Lewis, P = 0.0555<br>4T1 vs. MC38, P = 0.2876<br>Lewis vs. MC38, P > 0.9999<br>**18:**<br>4T1 vs. Lewis, P = 0.0024<br>4T1 vs. MC38, P = 0.0194<br>Lewis vs. MC38, P > 0.9999<br>**21:**<br>4T1 vs. Lewis, P > 0.9999<br>4T1 vs. MC38, P > 0.9999<br>Lewis vs. MC38, P > 0.9999	**9:**<br>4T1 = 16.716 ± 8.301<br>Lewis = 33.458 ± 11.854<br>MC38 = 40.474 ± 26.104<br>**12:**<br>4T1 = 79.230 ± 28.662<br>Lewis = 149.382 ± 25.863<br>MC38 = 102.259 ± 28.193<br>**15:**<br>4T1 = 289.421 ± 74.240<br>Lewis = 548.365 ± 78.258<br>MC38 = 471.501 ± 48.497<br>**18:**<br>4T1 = 661.933 ± 80.847<br>Lewis = 1034.424 ± 113.907<br>MC38 = 970.463 ± 96.327<br>**21:**<br>4T1 = 1310.960 ± 149.848<br>Lewis = 1372.199 ± 106.242<br>MC38 = 1396.151± 107.554
**1C**	**One-way ANOVA**	**Dunnett's multiple comparisons test**	PBS = 79.80 ± 1.010<br>4T1 = 41.41 ± 4.909<br>Lewis = 64.24 ± 5.595
PBS = 10	F (3, 37) = 14.03	PBS vs. 4T1, P <	

4T1 = 11<br>Lewis = 10<br>MC38 = 10	P < 0.0001	0.0001<br>PBS vs. Lewis, P = 0.0442<br>PBS vs. MC38, P = 0.1295	MC38 = 67.35 ± 4.221
**1D**	**One-way ANOVA**	**Dunnett's multiple comparisons test**	PBS = 95.53 ± 14.98<br>4T1 = 152.7 ± 12.26<br>Lewis = 156.6 ± 14.12<br>MC38 = 147.6 ±13.40
PBS = 10<br>4T1 = 11<br>Lewis = 10<br>MC38 = 9	F (3, 36) = 4.382<br>P = 0.0100	PBS vs. 4T1, P = 0.0126<br>PBS vs. Lewis, P = 0.0091<br>PBS vs. MC38, P = 0.0344	
**1E**	**One-way ANOVA**	**Dunnett's multiple comparisons test**	PBS = 49.67 ± 9.116<br>4T1 = 125.55 ± 9.861<br>Lewis = 105.6 ± 8.884<br>MC38 = 105.8 ± 11.75
PBS = 10<br>4T1 = 11<br>Lewis = 9<br>MC38 = 10	F (3, 36) = 11.27<br>P < 0.0001	PBS vs. 4T1, P < 0.0001<br>PBS vs. Lewis, P = 0.0014<br>PBS vs. MC38, P = 0.0010	
**1F**	**One-way ANOVA**	**Dunnett's multiple comparisons test**	PBS = 32.78 ± 2.593<br>4T1 = 29.73 ± 2.381<br>Lewis = 32.71 ± 2.950<br>MC38 = 28.68 ± 1.933
PBS = 11<br>4T1 = 10<br>Lewis = 10<br>MC38 = 10	F (3, 37) = 0.6978<br>P = 0.5594	PBS vs. 4T1, P = 0.7200<br>PBS vs. Lewis, P > 0.9999<br>PBS vs. MC38, P = 0.5174	
**1H**	**Two-way ANOVA**	**Bonferroni's multiple comparisons test**	Vehicle + PBS = 15.32 ± 2.961<br>Vehicle + 4T1 = 26.000 ± 3.024<br>GW4869 + PBS = 4.940 ± 0.470<br>GW4869 + 4T1 = 7.048 ± 0.519
Vehicle + PBS = 4<br>Vehicle + 4T1 = 4<br>GW4869 + PBS = 4<br>GW4869 + 4T1 = 4	GW4869: F (1, 12) = 46.75, P < 0.0001<br>4T1: F (1, 12) = 8.885, P = 0.0115<br>GW4869 × 4T1: F (1, 12) = 3.993, P = 0.0689	Vehicle + PBS vs. Vehicle + 4T1, P = 0.0084<br>GW4869 + PBS vs. GW4869 + 4T1, P >0.9999<br>Vehicle + PBS vs. GW4869 + PBS, P = 0.0101<br>Vehicle + 4T1 vs. GW4869 + 4T1, P < 0.0001.	

**1I**	**Two-way ANOVA**	**Bonferroni's multiple comparisons test**	Vehicle + PBS = 491.500 ± 43.119<br>Vehicle + 4T1 = 734.206 ± 47.781<br>GW4869 + PBS = 225.004 ± 20.813<br>GW4869 + 4T1 = 334.398 ± 10.824
Vehicle + PBS = 4<br>Vehicle + 4T1 = 4<br>GW4869 + PBS = 4<br>GW4869 + 4T1 = 4	GW4869: F (1, 12) = 94.61, P < 0.0001<br>4T1: F (1, 12) = 26.42, P = 0.0002<br>GW4869 × 4T1: F (1, 12) = 3.787, P = P=0.0754	Vehicle + PBS vs. Vehicle + 4T1, P = 0.0006<br>GW4869 + PBS vs. GW4869 + 4T1, P = 0.0867<br>Vehicle + PBS vs. GW4869 + PBS, P = 0.0003<br>Vehicle + 4T1 vs. GW4869 + 4T1, P < 0.0001.	
**1J**	**Two-way ANOVA**	**Bonferroni's multiple comparisons test**	Vehicle + PBS = 82.89 ± 1.60<br>Vehicle + 4T1 = 62.96 ± 2.86<br>GW4869 + PBS = 82.67 ± 1.32<br>GW4869 + 4T1 = 80.35 ± 2.06
Vehicle + PBS = 12<br>Vehicle + 4T1 = 13<br>GW4869 + PBS = 12<br>GW4869 + 4T1 = 13	GW4869: F (1, 46) = 16.920, P = 0.0002<br>4T1: F (1, 46) = 28.360, P < 0.0001<br>GW4869 × 4T1: F (1, 46) = 17.770, P = 0.0001	Vehicle + PBS vs. Vehicle + 4T1, P < 0.0001.<br>GW4869 + PBS vs. GW4869 + 4T1, P =0.8725<br>Vehicle + PBS vs. GW4869 + PBS, P > 0.9999<br>Vehicle + 4T1 vs. GW4869 + 4T1, P < 0.0001.	
**1K**	**Two-way ANOVA**	**Bonferroni's multiple comparisons test**	Vehicle + PBS = 136.17 ± 6.11<br>Vehicle + 4T1 = 184.92 ± 6.10<br>GW4869 + PBS = 138.17 ± 7.19<br>GW4869 + 4T1 = 144.85 ± 8.10
Vehicle + PBS = 12<br>Vehicle + 4T1 = 13<br>GW4869 + PBS = 12<br>GW4869 + 4T1 = 13	GW4869: F (1, 46) = 7.179, P = 0.0102<br>4T1: F (1, 46) = 15.220, P = 0.0003<br>GW4869 × 4T1: F (1, 46) = 8.766, P = 0.0048	Vehicle + PBS vs. Vehicle + 4T1, P < 0.0001<br>GW4869 + PBS vs. GW4869 + 4T1, P > 0.9999<br>Vehicle + PBS vs. GW4869 + PBS, P > 0.9999<br>Vehicle + 4T1 vs. GW4869 + 4T1, P =	

		0.0004.	
**1L**	**Two-way ANOVA**	**Bonferroni's multiple comparisons test**	Vehicle + PBS = 75.78 ± 4.43<br>Vehicle + 4T1 = 126.73 ± 7.13<br>GW4869 + PBS = 94.42 ± 8.41<br>GW4869 + 4T1 = 91.55 ± 6.20
Vehicle + PBS = 9<br>Vehicle + 4T1 = 11<br>GW4869 + PBS = 12<br>GW4869 + 4T1 = 11	GW4869: F (1, 39) = 1.379, P = 0.2474<br>4T1: F (1, 39) = 11.65, P = 0.0015<br>GW4869 × 4T1: F (1, 39) = 14.59, P = 0.0005	Vehicle + PBS vs. Vehicle + 4T1, P < 0.0001<br>GW4869 + PBS vs. GW4869 + 4T1, P > 0.9999<br>Vehicle + PBS vs. GW4869 + PBS, P = 0.1467<br>Vehicle + 4T1 vs. GW4869 + 4T1, P = 0.0018	
**1M**	**Two-way ANOVA**	**Bonferroni's multiple comparisons test**	
Vehicle + PBS = 12<br>Vehicle + 4T1 = 13<br>GW4869 + PBS = 12<br>GW4869 + 4T1 = 13	GW4869: F (1, 46) = 0.003, P = 0.9541<br>4T1: F (1, 46) = 0.5608, P = 0.4577<br>GW4869 × 4T1: F (1, 46) = 0.1078, P = 0.7441	Vehicle + PBS vs. Vehicle + 4T1, P = 0.9002<br>GW4869 + PBS vs. GW4869 + 4T1, P = P > 0.9999<br>Vehicle + PBS vs. GW4869 + PBS, P > 0.9999<br>Vehicle + 4T1 vs. GW4869 + 4T1, P > 0.9999	Vehicle + PBS = 50.75 ± 4.47<br>Vehicle + 4T1 = 54.53 ± 2.46<br>GW4869 + PBS = 51.70 ± 4.49<br>GW4869 + 4T1 = 53.17 ± 2.31
**1O**	**Unpaired t test**		PBS = 78.01 ± 2.410<br>4T1-EVs = 69.78 ± 2.35
PBS = 12<br>4T1-EVs = 13	t = 2.444,<br>P = 0.0226		
**1P**	**Unpaired t test**		PBS = 132.67 ± 8.92<br>4T1-EVs = 179.92 ± 6.89
PBS = 12<br>4T1-EVs = 13	t = 4.230,<br>P = 0.0003		
**1Q**	**Unpaired t test**		PBS = 84.67 ± 8.89<br>4T1-EVs = 138.15 ± 10.96
PBS = 12<br>4T1-EVs = 13	t = 3.752,<br>P = 0.0010		
**1R**	**Unpaired t test**		PBS = 42.02 ± 1.54<br>4T1-EVs = 43.91 ± 2.14
PBS = 12<br>4T1-EVs = 13	t = 0.708<br>P = 0.4861		
**2A**	**One-way ANOVA**	**Bonferroni's**	PBS+Vehicle = 1.025 ±

		**multiple comparisons test**	0.153<br>4T1+Vehicle = 3.619± 0.577<br>4T1+RNase = 3.326 ± 0.585<br>4T1+RNase+Triton-100 = 0.000 ± 0.000
PBS+Vehicle = 5<br>4T1+Vehicle = 5<br>4T1+RNase = 5<br>4T1+RNase+Triton-100 = 5	F (3, 16) = 17.81<br>P < 0.0001	PBS+Vehicle vs. 4T1+Vehicle, P = 0.0027<br>PBS+Vehicle vs. 4T1+RNase, P = 0.0077<br>4T1+Vehicle vs. 4T1+RNase P > 0.9999	
**2B**	**Unpaired t test**		mPFC:<br>PBS = 1.00 ± 0.06<br>4T1 = 7.90 ± 0.99<br>Amy:<br>PBS = 1.00 ± 0.09<br>4T1 = 1.03 ± 0.06<br>NAc:<br>PBS = 1.00 ± 0.06<br>4T1 =1.00 ± 0.07<br>Hip:<br>PBS = 1.00 ± 0.15<br>4T1 = 2.11 ± 0.25<br>VTA:<br>PBS = 1.00 ± 0.09<br>4T1 = 0.88 ± 0.09
mPFC:<br>PBS = 8<br>4T1 = 8<br>Amy:<br>PBS = 6<br>4T1 = 6<br>NAc:<br>PBS = 7<br>4T1 = 7<br>Hip:<br>PBS = 8<br>4T1 = 8<br>VTA:<br>PBS = 6<br>4T1 = 6	mPFC:<br>t = 6.922<br>P < 0.0001<br>Amy:<br>t = 0.229<br>P = 0.8233<br>NAc:<br>t = 0.015<br>P = 0.9885<br>Hip:<br>t =3.825,<br>P = 0.0019<br>VTA:<br>t = 0.930,<br>P = 0.3741		
**2C**	**Unpaired t test**		PPAR-δ:<br>PBS= 1.00 ± 0.14<br>4T1 = 0.50 ± 0.07<br><br>Twist1：<br>PBS = 1.00 ± 0.13<br>4T1= 1.76 ± 0.13
PBS + Twist1 = 11<br>PBS + PPAR-δ = 11<br>4T1 + Twist1 =11<br>4T1 + PPAR-δ = 11	PPAR-δ:<br>t = 3.196<br>P = 0.0045<br><br>Twist1：<br>t = 4.181<br>P = 0.0005		
**2E**	**Two-way ANOVA**	**Bonferroni's multiple comparisons test**	
**Twist1:**<br>PBS + LV-shCon =10<br>PBS + LV-shTwist1 = 10<br>4T1 + LV-shCon = 10	**Twist1:**<br>LV-shTwist1: F (1, 36) = 163.8, P < 0.0001<br>4T1: F (1, 36) =	**Twist1:**<br>PBS + LV-shCon vs. PBS + LV-shTwist1, P = 0.0037<br>4T1 + LV-shCon vs.	**Twist1:**<br>PBS + LV-shCon = 1.00 ± 0.082<br>PBS + LV-shTwist1 = 0.56 ± 0.071

4T1 + LV-shTwist1 = 10<br>**PPAR-δ:**<br>PBS + LV-shCon =10<br>PBS + LV-shTwist1 = 10<br>4T1 + LV-shCon = 10<br>4T1 + LV-shTwist1 = 10	53.22, P < 0.0001<br>LV-shTwist1 × 4T1: F (1, 36) = 6.445, P = 0.0156<br>**PPAR-δ:**<br>LV-shTwist1: F (1, 36) = 21.53, P < 0.0001<br>4T1: F (1, 36) = 6.149, P = 0.0180<br>LV-shTwist1 × 4T1: F (1, 36) = 1.661, P = 0.2057	4T1 + LV-shTwist1, P < 0.0001<br>PBS + LV-shCon vs. 4T1 + LV-shCon, P < 0.0001<br>PBS + LV-shTwist1 vs. 4T1 + LV-shTwist1, P < 0.0001<br>**PPAR-δ:**<br>PBS + LV-shCon vs. PBS + LV-shTwist1, P = 0.0466<br>4T1 + LV-shCon vs. 4T1 + LV-shTwist1, P = 0.0003<br>PBS + LV-shCon vs. 4T1 + LV-shCon, P = 0.0229<br>PBS + LV-shTwist1 vs. 4T1 + LV-shTwist1, P = 0.8107	4T1 + LV-shCon = 7.75 ± 0.762<br>4T1 + LV-shTwist1 = 2.37 ± 0.500<br>**PPAR-δ:**<br>PBS + LV-shCon = 1.00 ± 0.074<br>PBS + LV-shTwist1 = 1.27 ± 0.045<br>4T1 + LV-shCon = 0.69 ± 0.044<br>4T1 + LV-shTwist1 = 1.18 ± 0.131
**2F**	**Two-way ANOVA**	**Bonferroni's multiple comparisons test**	PBS + LV-shCon = 83.056 ± 2.167<br>PBS + LV-shTwist1 = 87.268 ± 1.619<br>4T1 + LV-shCon = 65.144 ± 3.352<br>4T1 + LV-shTwist1 = 79.505 ± 2.742
PBS + LV-shCon =10<br>PBS + LV-shTwist1 = 10<br>4T1 + LV-shCon = 10<br>4T1 + LV-shTwist1 = 10	LV-shTwist1: F (1, 36) = 13.23, P < P = 0.0009<br>4T1: F (1, 36) = 25.29, P < 0.0001<br>LV-shTwist1 × 4T1: F (1, 36) = 3.952, P = 0.0545	PBS + LV-shCon vs. PBS + LV-shTwist1, P = 0.5021<br>4T1 + LV-shCon vs. 4T1 + LV-shTwist1, P = 0.0006<br>PBS + LV-shCon vs. 4T1 + LV-shCon, P < 0.0001<br>PBS + LV-shTwist1 vs. 4T1 + LV-shTwist1, P = 0.0766	
**2G**	**Two-way ANOVA**	**Bonferroni's multiple comparisons test**	PBS + LV-shCon = 117.77 ± 13.122<br>PBS + LV-shTwist1 = 129.25 ± 13.477<br>4T1 + LV-shCon =
PBS + LV-shCon =10<br>PBS + LV-shTwist1 =	LV-shTwist1: F (1, 36) = 4.113, P =	PBS + LV-shCon vs. PBS + LV-shTwist1, P	

10<br>4T1 + LV-shCon = 10<br>4T1 + LV-shTwist1 = 10	0.0500<br>4T1: F (1, 36) = 5.435, P = 0.0255<br>LV-shTwist1 × 4T1: F (1, 36) = 9.369, P = 0.0042	= 0.9398<br>4T1 + LV-shCon vs. 4T1 + LV-shTwist1, P = 0.0019<br>PBS + LV-shCon vs. 4T1 + LV-shCon, P = 0.0010<br>PBS + LV-shTwist1 vs. 4T1 + LV-shTwist1, P > 0.9999	177.70 ± 9.154<br>4T1 + LV-shTwist1 = 121.140 ± 7.516
**2H**	**Two-way ANOVA**	**Bonferroni's multiple comparisons test**	PBS + LV-shCon = 113.2 ± 13.712<br>PBS + LV-shTwist1 = 122.5 ± 11.455<br>4T1 + LV-shCon = 164.5 ± 11.301<br>4T1 + LV-shTwist1 = 116.4 ± 12.571
PBS + LV-shCon =10<br>PBS + LV-shTwist1 = 10<br>4T1 + LV-shCon = 10<br>4T1 + LV-shTwist1 = 10	LV-shTwist1: F (1, 36) = 2.488, P = 0.1234<br>4T1: F (1, 36) = 3.377, P = 0.0744<br>LV-shTwist1 × 4T1: F (1, 36) = 5.446, P = 0.0253	PBS + LV-shCon vs. PBS + LV-shTwist1, P > 0.9999<br>4T1 + LV-shCon vs. 4T1 + LV-shTwist1, P = 0.0178<br>PBS + LV-shCon vs. 4T1 + LV-shCon, P = 0.0111<br>PBS + LV-shTwist1 vs. 4T1 + LV-shTwist1, P > 0.9999	
**2I**	**Two-way ANOVA**	**Bonferroni's multiple comparisons test**	PBS + LV-shCon = 28.321 ± 1.559<br>PBS + LV-shTwist1 = 25.743 ± 2.115<br>4T1 + LV-shCon = 26.769 ± 1.404<br>4T1 + LV-shTwist1 = 24.304 ± 1.860
PBS + LV-shCon =10<br>PBS + LV-shTwist1 = 10<br>4T1 + LV-shCon = 10<br>4T1 + LV-shTwist1 = 10	LV-shTwist1: F (1, 36) = 2.061, P = 0.1597<br>4T1: F (1, 36) = 0.7256, P = P = 0.3999<br>LV-shTwist1 × 4T1: F (1, 36) = 0.001, P = P=0.9745	PBS + LV-shCon vs. PBS + LV-shTwist1, P = 0.6124<br>4T1 + LV-shCon vs. 4T1 + LV-shTwist1, P = 0.6551<br>PBS + LV-shCon vs. 4T1 + LV-shCon, P > 0.9999<br>PBS + LV-shTwist1 vs. 4T1 + LV-shTwist1, P > 0.9999	
**3B**	**Unpaired t test**		4T1/shCon = 1.00 ±

4T1/shCon = 12<br>4T1/shTwist1 = 13	t = 7.910<br>P < 0.0001		0.10<br>4T1/shTwist1 = 0.21 ± 0.02
**3C**	**Unpaired t test**		4T1/shCon = 1.00 ± 0.14<br>4T1/shTwist1 = 0.23 ± 0.05
4T1/shCon = 12<br>4T1/shTwist1 = 14	t = 5.380<br>P < 0.0001		
**3D**	**Unpaired t test**		4T1/shCon = 1.00 ± 0.03<br>4T1/shTwist1 = 0.29 ± 0.02
4T1/shCon = 12<br>4T1/shTwist1 = 12	t = 22.519<br>P < 0.0001		
**3E**	**Two-way ANOVA**	**Bonferroni's multiple comparisons test**	4T1/shCon + Day 9 = 5.87 ± 3.13<br>4T1/shCon + Day 12 = 26.69 ± 3.57<br>4T1/shCon + Day 15 = 152.52 ± 21.98<br>4T1/shCon + Day 18 = 518.57 ± 43.50<br>4T1/shCon + Day 18 = 1022.27 ± 94.00<br>4T1/shTwist1 + Day 9 = 5.87 ± 3.13<br>4T1/shTwist1 + Day 12 = 22.81 ± 3.68<br>4T1/shTwist1 + Day 15 = 34.75 ± 3.91<br>4T1/shTwist1 + Day 18 = 60.59 ± 7.52<br>4T1/shTwist1 + Day 21 = 126.83 ± 26.21
4T1/shCon = 10<br>4T1/shTwist1 = 10	Days: F (4, 90) = 94.87, P < 0.0001<br>shTwist1: F (1, 90) = 181.1, P < 0.0001<br>Days × shTwist1: F (4, 90) = 61.41, P < 0.0001	4T1/shCon + Day 9 vs. 4T1/shTwist1 + Day 9, P > 0.9999<br>4T1/shCon + Day 12 vs. 4T1/shTwist1 + Day 12, P > 0.9999<br>4T1/shCon + Day 15 vs. 4T1/shTwist1 + Day 15, P = 0.0917<br>4T1/shCon + Day 18 vs. 4T1/shTwist1 + Day 18, P < 0.0001<br>4T1/shCon + Day 21 vs. 4T1/shTwist1 + Day 21, P < 0.0001	
**3F**	**One-way ANOVA**	**Bonferroni's multiple comparisons test**	Control = 86.63 ± 1.64<br>4T1/shCon = 66.01 ± 3.61<br>4T1/shTwist1 = 85.52 ± 1.80
Control = 10<br>4T1/shCon = 11<br>4T1/shTwist1 = 11	F (2, 29) = 20.757<br>P < 0.0001	Control vs. 4T1/shCon, P < 0.0001<br>Control vs. 4T1/shTwist1, P > 0.9999<br>4T1/shCon vs. 4T1/shTwist1, P < 0.0001	
**3G**	**One-way ANOVA**	**Bonferroni's**	Control = 121.40 ± 7.72

		**multiple comparisons test**	4T1/shCon = 169.00 ± 11.31<br>4T1/shTwist1 = 112.55 ± 7.70
Control = 10<br>4T1/shCon = 11<br>4T1/shTwist1 = 11	F (2, 29) = 11.235<br>P = 0.0002	4T1/shCon vs. Control, P = 0.0031<br>Control vs. 4T1/shTwist1, P > 0.9999<br>4T1/shCon vs. 4T1/shTwist1, P = 0.0004	
**3H**	**One-way ANOVA**	**Bonferroni's multiple comparisons test**	Control = 89.20 ± 7.76<br>4T1/shCon = 153.73 ± 9.22<br>4T1/shTwist1 = 112.64 ± 9.75
Control = 10<br>4T1/shCon = 11<br>4T1/shTwist1 = 11	F (2, 29) = 12.990<br>P < 0.0001	Control vs. 4T1/shCon, P < 0.0001<br>Control vs. 4T1/shTwist1, P = 0.2377<br>4T1/shCon vs. 4T1/shTwist1, P = 0.0084	
**3I**	**One-way ANOVA**	**Bonferroni's multiple comparisons test**	Control = 24.52 ± 2.05<br>4T1/shCon = 23.46 ± 1.24<br>4T1/shTwist1 = 24.88 ± 1.45
Control = 10<br>4T1/shCon = 11<br>4T1/shTwist1 = 11	F (2, 29) = 0.223<br>P = 0.8013	Control vs. 4T1/shCon, P > 0.9999<br>Control vs. 4T1/shTwist1, P > 0.9999<br>4T1/shCon vs. 4T1/shTwist1, P > 0.9999	
**3J**	**One-way ANOVA**	**Bonferroni's multiple comparisons test**	Control = 1.00 ± 0.07<br>4T1/shCon = 3.21 ± 0.22<br>4T1/shTwist1 = 0.94 ± 0.09
Control = 10<br>4T1/shCon = 10<br>4T1/shTwist1 = 10	F (2, 27) = 80.409<br>P < 0.0001	Control vs. 4T1, P < 0.0001<br>4T1/shCon vs. 4T1/shTwist1, P < 0.0001	
**3K**	**One-way ANOVA**	**Bonferroni's multiple**	

		**comparisons test**	Control = 1.00 ± 0.15<br>4T1/shCon = 5.64 ± 1.10<br>4T1/shTwist1 = 2.38 ± 0.66<br><br>Control = 1.00 ± 0.10<br>4T1/shCon = 0.51 ± 0.07<br>4T1/shTwist1 = 0.78 ± 0.06
Control = 10<br>4T1/shCon = 11<br>4T1/shTwist1 = 11	**Twist1:**<br>F (2, 29) = 9.558<br>P = 0.0006<br><br>**PPAR-δ:**<br>F (2, 29) = 10.26<br>P = 0.0004	Control vs. 4T1/shCon, P = 0.0006<br>Control vs. 4T1/shTwist1, P = 0.6532<br>4T1/shCon vs. 4T1/shTwist1, P = 0.0149<br><br>Control vs. 4T1/shCon, P = 0.0003<br>Control vs. 4T1/shTwist1, P = 0.1662<br>4T1/shCon vs. 4T1/shTwist1, P = 0.0462	
**4B**	**One-way ANOVA**	**Bonferroni's multiple comparisons test**	
PBS-SDEVs = 13<br>4T1/shCon-SDEVs = 14<br>4T1/shTwist1-SDEVs = 13	F (2, 37) = 12.131<br>P < 0.0001	PBS-SDEVs vs. 4T1/shCon-SDEVs, P = 0.0005<br>PBS-SDEVs vs. 4T1/shTwist1-SDEVs, P > 0.9999<br>4T1/shCon-SDEVs vs. 4T1/shTwist1-SDEVs, P = 0.0004	PBS-SDEVs = 78.31 ± 2.36<br>4T1/shCon-SDEVs = 64.42 ± 2.53<br>4T1/shTwist1-SDEVs = 78.56 ± 2.10
**4C**	**One-way ANOVA**	**Bonferroni's multiple comparisons test**	PBS-SDE = 133.38 ± 10.50<br>4T1/shCon-SDE = 187.43 ± 7.77<br>4T1/shTwist1-SDE = 154.85 ± 8.31
PBS-SDEVs = 13<br>4T1/shCon-SDEVs = 14<br>4T1/shTwist1-SDEVs = 13	F (2, 37) = 9.532<br>P = 0.0005	PBS-SDE vs. 4T1/shCon-SDE, P = 0.0003<br>PBS-SDE vs. 4T1/shTwist1-SDE, P =<br>0.3003<br>4T1/shCon-SDE vs.	

		4T1/shTwist1-SDE, P = 0.0392	
**4D**	**One-way ANOVA**	**Bonferroni's multiple comparisons test**	PBS-SDE = 79.15 ± 9.02<br>4T1/shCon-SDE = 128.00 ± 12.23<br>4T1/shTwist1-SDE = 88.77 ± 9.35
PBS-SDEVs = 13<br>4T1/shCon-SDEVs = 14<br>4T1/shTwist1-SDEVs = 13	F (2, 37) = 6.304<br>P = 0.0044	PBS-SDEVs vs. 4T1/shCon-SDE, P = 0.0059<br>PBS-SDE vs. 4T1/shTwist1-SDE, P > 0.9999<br>4T1/shCon-SDE vs. 4T1/shTwist1-SDE, P = 0.0330	
**4E**	**One-way ANOVA**	**Bonferroni's multiple comparisons test**	PBS-SDE = 42.03 ± 2.36<br>4T1/shCon-SDE = 47.25 ± 2.01<br>4T1/shTwist1-SDE = 39.92 ± 1.98
PBS-SDEVs = 13<br>4T1/shCon-SDEVs = 14<br>4T1/shTwist1-SDEVs = 13	F (2, 37) = 3.239<br>P = 0.0506	PBS-SDE vs. 4T1/shCon-SDE, P = 0.2649<br>PBS-SDE vs. 4T1/shTwist1-SDE, P > 0.9999<br>4T1/shCon-SDE vs. 4T1/shTwist1-SDE, P = 0.0558	
**4F**	**One-way ANOVA**	**Bonferroni's multiple comparisons test**	PBS-SDE = 1.00 ± 0.05<br>4T1/shCon-SDE = 2.57 ± 0.10<br>4T1/shTwist1-SDE = 0.91 ± 0.06
PBS-SDEVs = 10<br>4T1/shCon-SDEVs = 12<br>4T1/shTwist1-SDEVs = 10	F (2, 29) = 153.705<br>P < 0.0001	PBS-SDE vs. 4T1/shCon-SDE, P < 0.0001<br>PBS-SDE vs. 4T1/shTwist1-SDE, P > 0.9999<br>4T1/shCon-SDE vs. 4T1/shTwist1-SDE, P < 0.0001	
**4G**	**One-way ANOVA**	**Bonferroni's multiple comparisons test**	PBS-SDE = 1.00 ± 0.10<br>4T1/shCon-SDE = 2.44 ± 0.26<br>4T1/shTwist1-SDE = 1.00 ± 0.14
PBS-SDEVs = 8<br>4T1/shCon-SDEVs = 8<br>4T1/shTwist1-SDEVs =	F (2, 21) = 20.986<br>P < 0.0001	PBS-SDE vs. 4T1/shCon-SDE, P < 0.0001	

8		PBS-SDE vs. 4T1/shTwist1-SDE, P > 0.9999. 4T1/shCon-SDE vs. 4T1/shTwist1-SDE, P < 0.0001	
**4I**	**One-way ANOVA**	**Bonferroni's multiple comparisons test**	PBS-SDE = 81.57 ± 1.97<br>4T1/shCon-SDE = 65.53 ± 2.91<br>4T1/shTwist1-SDE = 79.94 ± 1.88
PBS-SDEVs = 13<br>4T1/shCon-SDEVs = 13<br>4T1/shTwist1-SDEVs = 13	F (2, 36) = 14.735<br>P < 0.0001	PBS-SDEVs vs. 4T1/shCon-SDEVs, P < 0.0001<br>PBS-SDEVs vs. 4T1/shTwist1-SDEVs, P > 0.9999<br>4T1/shCon-SDEVs vs. 4T1/shTwist1-SDEVs, P = 0.0003	
**4J**	**One-way ANOVA**	**Bonferroni's multiple comparisons test**	PBS-SDE = 128.54 ± 8.87<br>4T1/shCon-SDE = 167.92 ± 8.62<br>4T1/shTwist1-SDE = 152.00 ± 8.69
PBS-SDEVs = 13<br>4T1/shCon-SDEVs = 13<br>4T1/shTwist1-SDEVs = 13	F (2, 36) = 5.152<br>P = 0.0108	4T1/shCon-SDE vs. PBS-SDE, P = 0.0088<br>4T1/shCon-SDE vs. 4T1/shTwist1-SDE, P = 0.6159	
**4K**	**One-way ANOVA**	**Bonferroni's multiple comparisons test**	PBS-SDE = 87.67 ± 9.24<br>4T1/shCon-SDE = 134.92 ± 8.11<br>4T1/shTwist1-SDE = 95.25 ± 8.40
PBS-SDEVs = 12<br>4T1/shCon-SDEVs = 13<br>4T1/shTwist1-SDEVs = 12	F (2, 34) = 8.969<br>P = 0.0007	4T1/shCon-SDE vs. PBS-SDE, P = 0.0012<br>4T1/shCon-SDE vs. 4T1/shTwist1-SDE, P = 0.0070	
**4L**	**One-way ANOVA**	**Bonferroni's multiple comparisons test**	PBS-SDE = 49.61 ± 3.37<br>4T1/shCon-SDE = 46.76 ± 2.22<br>4T1/shTwist1-SDE = 46.87 ± 4.07
PBS-SDEVs = 13<br>4T1/shCon-SDEVs = 13<br>4T1/shTwist1-SDEVs = 13	F (2, 36) = 0.2388<br>P = 0.7888	PBS-SDE vs. 4T1/shCon-SDE, P > 0.9999<br>PBS-SDE vs. 4T1/shTwist1-SDE, P > 0.9999	

**4M**	**One-way ANOVA**	**Bonferroni's multiple comparisons test**	PBS-SDE = 1.00 ± 0.09<br>4T1/shCon-SDE = 1.86 ± 0.18<br>4T1/shTwist1-SDE = 0.81 ± 0.11
PBS-SDE = 10<br>4T1/shCon-SDE = 12<br>4T1/shTwist1-SDE = 10	F (2, 29) = 16.470<br>P < 0.0001	PBS-SDE vs. 4T1/shCon-SDE, P = 0.0005<br>PBS-SDE vs. 4T1/shTwist1-SDE, P > 0.9999<br>4T1/shCon-SDE vs. 4T1/shTwist1-SDE, P < 0.0001	
**4N**	**One-way ANOVA**	**Bonferroni's multiple comparisons test**	PBS-SDE = 1.00 ± 0.21<br>4T1/shCon-SDE = 1.82 ± 0.23<br>4T1/shTwist1-SDE = 0.87 ± 0.09
PBS-SDE = 8<br>4T1/shCon-SDE = 8<br>4T1/shTwist1-SDE = 8	F (2, 21) = 7.760<br>P = 0.0030	PBS-SDE vs. 4T1/shCon-SDE, P = 0.0147<br>PBS-SDE vs. 4T1/shTwist1-SDE, P > 0.9999<br>4T1/shCon-SDE vs. 4T1/shTwist1-SDE, P = 0.0047	
**5D**	**One-way ANOVA**	**Bonferroni's multiple comparisons test**	
PBS-SDEVs = 20<br>4T1/shCon-SDEVs = 20<br>4T1/shTwist1-SDEVs = 20	F (2, 57) = 20.651<br>P < 0.0001	PBS-SDEVs vs. 4T1/shCon-SDEVs, P < 0.0001<br>PBS-SDEVs vs. 4T1/shTwist1-SDEVs, P = 0.0984<br>4T1/shCon-SDEVs vs. 4T1/shTwist1-SDEVs, P = 0.0003	PBS-SDEVs = 23.95 ± 1.05<br>4T1/shCon-SDEVs = 14.55 ± 1.12<br>4T1/shTwist1-SDEVs = 20.70 ± 0.98
**5E**	**One-way ANOVA**	**Bonferroni's multiple comparisons test**	PBS-SDEVs = 2785.30 ± 113.55<br>4T1/shCon-SDEVs = 2094.00 ± 70.57<br>4T1/shTwist1-SDEVs = 2770.00 ± 94.61
PBS-SDEVs = 20<br>4T1/shCon-SDEVs = 20<br>4T1/shTwist1-SDEVs =	F (2, 57) = 17.430<br>P < 0.0001	PBS-SDEVs vs. 4T1/shCon-SDEVs, P < 0.0001<br>PBS-SDEVs vs.	

20		4T1/shTwist1-SDEVs, P > 0.9999<br>4T1/shCon-SDEVs vs. 4T1/shTwist1-SDEVs, P < 0.0001	
**5I**	**One-way ANOVA**	**Bonferroni's multiple comparisons test**	PBS-EVs = 17.97 ± 1.30<br>4T1/shCon-EVs = 12.38 ± 0.74<br>4T1/shTwist1-EVs = 16.62 ± 1.06
PBS-EVs = 29<br>4T1/shCon-EVs = 29<br>4T1/shTwist1-EVs = 29	F (2, 84) = 7.589<br>P = 0.0009	PBS-EVs vs. 4T1/shCon-EVs, P = 0.0010<br>PBS-EVs vs. 4T1/shTwist1-EVs, P > 0.9999<br>4T1/shCon-EVs vs. 4T1/shTwist1-EVs, P = 0.0173	
**5J**	**One-way ANOVA**	**Bonferroni's multiple comparisons test**	PBS-EVs = 1462.24 ± 58.11<br>4T1/shCon-EVs = 1162.48 ± 45.75<br>4T1/shTwist1-EVs = 1523.12 ± 51.45
PBS-EVs = 29<br>4T1/shCon-EVs = 29<br>4T1/shTwist1-EVs = 29	F (2, 84) = 13.777<br>P < 0.0001	PBS-EVs vs. 4T1/shCon-EVs, P = 0.0003<br>PBS-EVs vs. 4T1/shTwist1-EVs, P > 0.9999<br>4T1/shCon-EVs vs. 4T1/shTwist1-EVs, P < 0.0001	
**S2B**	**Unpaired t test**		PBS = 3.87 ± 0.27<br>GW4869 = 2.50 ± 0.11
PBS = 5<br>GW4869 = 5	t = 4.624<br>P = 0.0017		
**S3B**	**Unpaired t test**		PBS = 81.05 ± 1.40<br>4T1-EVs = 65.53 ± 2.74
PBS = 12<br>4T1-EVs = 14	t = 4.793<br>P < 0.0001		
**S3C**	**Unpaired t test**		PBS = 140.2 ± 12.63<br>4T1-EVs = 181.9 ± 10.94
PBS = 12<br>4T1-EVs = 14	t = 2.509<br>P = 0.019		
**S3D**	**Unpaired t test**		PBS = 87.83 ± 10.42<br>4T1-EVs = 135.9 ± 9.93
PBS = 12<br>4T1-EVs = 14	t = 3.334<br>P = 0.003		
**S3E**	**Unpaired t test**		PBS = 45.06 ± 2.17<br>4T1-EVs = 41.18 ±
PBS = 12	t = 1.200		

4T1-EVs = 14	P = 0.242		2.34
